# Cannabidiol-Mediated Changes to the Phospholipid Profile of UVB-Irradiated Keratinocytes from Psoriatic Patients

**DOI:** 10.3390/ijms21186592

**Published:** 2020-09-09

**Authors:** Wojciech Łuczaj, Izabela Dobrzyńska, Adam Wroński, M Rosário Domingues, Pedro Domingues, Elżbieta Skrzydlewska

**Affiliations:** 1 Department of Analytical Chemistry, Medical University of Bialystok, Mickiewicza 2d, 15-222 Bialystok, Poland; elzbieta.skrzydlewska@umb.edu.pl; 2 Faculty of Chemistry, University in Białystok, Ciołkowskiego 1K, 15-245 Białystok, Poland; izadob@uwb.edu.pl; 3 Dermatological Specialized Center “DERMAL” NZOZ in Bialystok, 15-453 Bialystok, Poland; adam.wronski@dermal.pl; 4 Department of Chemistry, Mass Spectrometry Center, LAQV, University of Aveiro, Campus Universitário de Santiago, 3810-193 Aveiro, Portugal; mrd@ua.pt (MR.D.); p.domingues@ua.pt (P.D.); 5 Department of Chemistry &, CESAM, University of Aveiro, Campus Universitário de Santiago, 3810-193 Aveiro, Portugal

**Keywords:** cannabidiol, lipidomics, keratinocytes, phospholipids, psoriasis vulgaris, UVB

## Abstract

UVB phototherapy is treatment for psoriasis, which increases phospholipid oxidative modifications in the cell membrane of the skin. Therefore, we carried out lipidomic analysis on the keratinocytes of healthy individuals and patients with psoriasis irradiated with UVB and treated with cannabidiol (CBD), phytocannabinoid with antioxidant and anti-inflammatory properties. Our results showed that, in psoriatic keratinocytes phosphatidylcholine (PC), phosphatidylinositol (PI), phosphatidylserine (PS), and ether-linked phosphoethanolamine (PEo), were downregulated, while SM (d41:2) was upregulated. These changes were accompanied by an increase in negative zeta potential, which indicates translocation of PS to the outer layer of the membrane. CBD treatment of psoriatic keratinocytes led to downregulation of PC, PS, and upregulation of certain PEo and an SM species, SM (d42:2), and the zeta potential. However, UVB irradiation of psoriatic keratinocytes resulted in upregulation of PC, PC plasmalogens (PCp), PEo, and a decrease in the negative zeta potential. The exposure of UVB-irradiated cells to CBD led to a decrease in the level of SM (d42:2). Our results suggest that CBD induces pro-apoptotic mechanisms in psoriatic keratinocytes while simultaneously improving the antioxidant properties and preventing the loss of transepidermal water of keratinocytes of patients irradiated with UVB. Thus, CBD has potential therapeutic value in the treatment of psoriasis.

## 1. Introduction

Phospholipids play a crucial role in the functioning of living organisms. In addition to their role as building blocks for cell membranes, they are also precursors of signaling molecules, including platelet-activating factor, inositol triphosphate, diacylglycerol, and lipid mediators such as leukotrienes, prostaglandins, and endocannabinoids [1]. Phospholipids and their metabolites have been found to participate in the regulation of inflammation and the immune response of the organism [2]. Thus, phospholipids have also recently emerged as key players in the development of skin diseases [3,4,5,6]. Psoriasis, after atopic dermatitis, is the most common chronic inflammatory skin disease, affecting approximately 4% of the population [7]. Psoriasis is characterized by a dysfunction of the skin barrier and a disruption of epidermal homeostasis. Several studies on psoriasis have shown systemic changes in the metabolism of phospholipids [6,8,9,10,11] and local changes in epidermal ceramides [12,13,14,15]. To date, the pathogenesis of this disease is not fully understood. However, it is well established that psoriasis is associated with a redox imbalance, which results in oxidative stress and oxidative modifications in cellular components, primarily phospholipids in the cell membrane [7]. Treatment for psoriasis usually involves the use of topical medications and UV therapy. However, the use of UVB radiation can also disturb the redox balance in cells, leading to a change in the metabolism of phospholipids [16,17,18]. Therefore, there is a need for new approaches to prevent such disorders of the metabolism of phospholipids, and natural compounds are being considered of particular interest.

One of the pharmacologically active phytocannabinoids found in *Cannabis sativa* L. is cannabidiol (CBD) [19]. CBD, which is not psychoactive, has antioxidant and anti-inflammatory properties and has been shown to improve symptoms of autoimmune diseases (including skin diseases like psoriasis) in animal models [20,21]. CBD restores redox imbalances, which result from medical conditions as well as the effects of physicochemical factors [16,22]. This effect is mediated by reducing the level of reactive oxygen species (ROS) and increasing the level and activity of endogenous antioxidants [16,22]. In addition, CBD together with vitamins A, E, C, and enzymes such as glutathione peroxidase and thioredoxin peroxidase, protects membrane phospholipids and prevents oxidation of non-enzymatic cellular antioxidants such as glutathione and thioredoxin [23]. Moreover, CBD reduces lipid peroxidation (measured as the levels of unsaturated α,β-reactive aldehydes) by preventing the generation of ROS and reducing the levels of enzymes involved in the metabolism of phospholipids. CBD also increases the level of endocannabinoids [16,22].

Although CBD has been shown to inhibit the proliferation of human keratinocytes [24,25], to date, no research has examined the effect of CBD on phospholipids in psoriatic keratinocytes, especially after exposure to UV radiation. Therefore, we investigated the impact of CBD on disease- and UVB-mediated changes in the phospholipid profile of keratinocytes of patients with psoriasis.

## 2. Results

In this work, we used a high-resolution HILIC-LC-MS/MS platform to characterize the changes in the phospholipid (PL) profile of keratinocytes of psoriatic patients with and without CBD and UVB treatment. In our samples, we identified PL species belonging to eight different classes. These were phosphatidylcholine (PC), phosphatidylethanolamine (PE), phosphatidylinositols (PI), phosphatidylserine (PS), lyso-PE (LPE), lyso-PC (LPC), cardiolipin (CL), and sphingomyelin (SM). The list of 119 PL species (corresponding to the most abundant species in all the classes identified) which were identified and quantified is presented in Supplementary Tables S1–S3.

Multivariate and univariate statistical analyses were used to identify significant changes in the profiles of phospholipids between groups. The data were first autoscaled and then subjected to a principal component analysis (PCA) to display the clustering trends of the experimental groups.

### 2.1. Comparison of the Phospholipid Profile of Healthy Keratinocytes and Psoriatic Keratinocytes Not Treated or Treated with CBD (Control vs. CBD vs. Ps vs. Ps + CBD)

**Figure 1:**
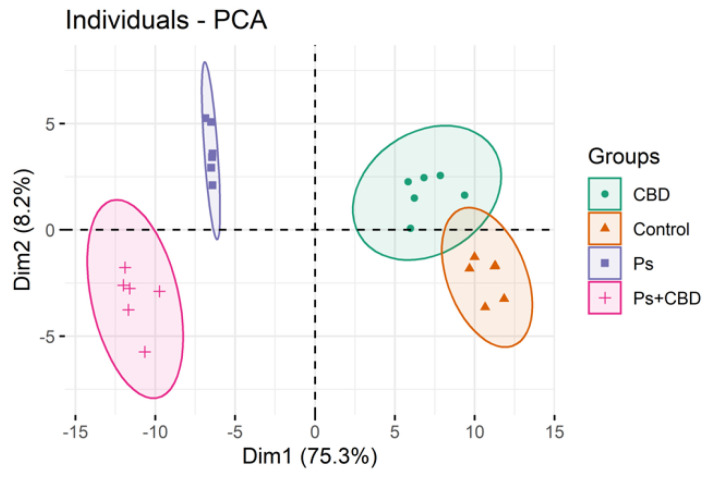
Two-dimensional principal component analysis (2D PCA) scores plot of the relative abundance of phospholipid species in keratinocytes, isolated from the skin of healthy subjects (Control) and psoriatic patients (Ps). These cells were not treated or treated with CBD (4 μM). The following groups of keratinocytes were examined: Control, CBD, Ps, and Ps + CBD. All samples were analyzed after 24 h of treatment.

We analyzed three data sets each comprising four groups. The first set included data from the following groups: control (healthy keratinocytes), CBD (healthy keratinocytes treated with CBD (4 μM)), Ps (psoriatic keratinocytes) and Ps + CBD (psoriatic keratinocytes treated with CBD (4 μM)). The two-dimensional principal component analysis (2D PCA) scores plot shows that the model captured 83.5% of the total variance in the dataset (Figure 1). The variation between the different biological groups is more pronounced along the Dim1 axis (75.3%), which counts for the greatest variation of the model. In this model, Dim2 (8.2%) describes mainly the variation within the groups, in particular in the groups of psoriatic keratinocytes. The PCA plot shows that the groups of psoriatic patients (treated or not treated with CBD) scattered in the left region of the plot were separated from the groups of healthy subjects (Control and CBD), which were scattered in the right region of the plot (Figure 1). Group separation was more pronounced between Ps and Ps + CBD than Control and CBD.

**Figure 2:**
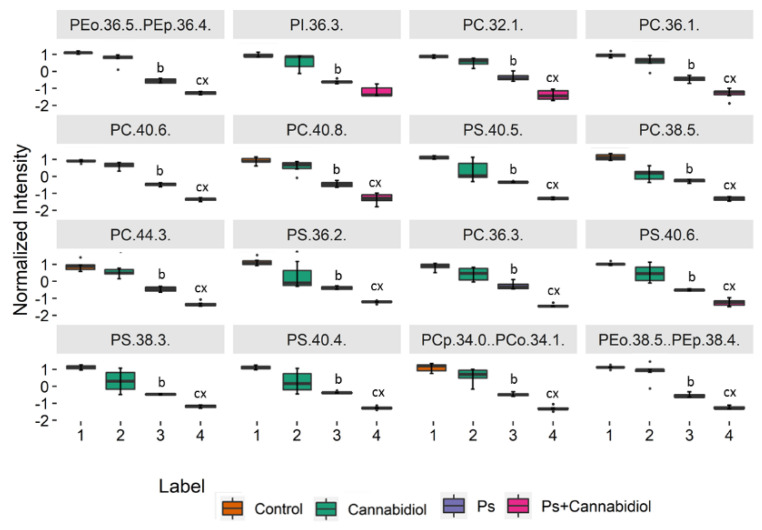
Boxplots of the 16 most discriminating PL molecular species (according to Kruskal–Wallis and the post hoc Dunn multiple comparison tests) identified in keratinocytes isolated from the skin of healthy subjects (Control) and psoriatic patients (Ps). These cells were either not treated or treated with Cannabidiol (CBD) (4 μM). The following groups of keratinocytes were studied: Control, CBD, Ps, and Ps + CBD. *p* < 0.05 was considered statistically significant: b, control vs. Ps; c − control vs. Ps + CBD; x − Ps vs. Ps + CBD.

Finally, for a more detailed interpretation of the data, we performed a univariate analysis (Kruskal–Wallis and the post hoc Dunn multiple comparison tests) in order to assess the variation of the relative abundance of the molecular species of phospholipids under the conditions studied (Figure 2, Table S4). The 16 most discriminating PL molecular species with a *p* < 0.05 are presented in Figure 2. We observed a statistically significant decrease in the levels of PC species (PC (32:1), PC (36:1), PC (40:6), PC (40:8), PC (38:5), PC (44:3), and PC (36:3)), PS species (PS (40:5), PS (36:2), PS (40:6), PS (38:3), and PS (40:4)), certain plasmalogens of PE species and PI (36:3) in psoriatic keratinocytes and psoriatic keratinocytes treated with CBD compared to control keratinocytes and control keratinocytes treated with CBD. We also observed a statistically significant decrease in all the above phospholipid species with exception of PI (36:3) in the keratinocytes of the Ps + CBD when compared to the Ps group (Figure 2).

To see if the 25 main discriminating phospholipid species, according to univariate analysis, allowed for discriminating between groups, we created a dendrogram with a two-dimensional hierarchical clustering (Figure S1). The primary split in the upper hierarchical dendrogram shows that the samples were clustered independently in the four main experimental groups and the clustering of the individual phospholipids shows that they clustered into one main group.

### 2.2. Comparison of the Phospholipid Profile of Healthy Keratinocytes Not Treated and Treated with CBD or/and UVB (Control vs. CBD vs. UVB vs. UVB + CBD)

**Figure 3:**
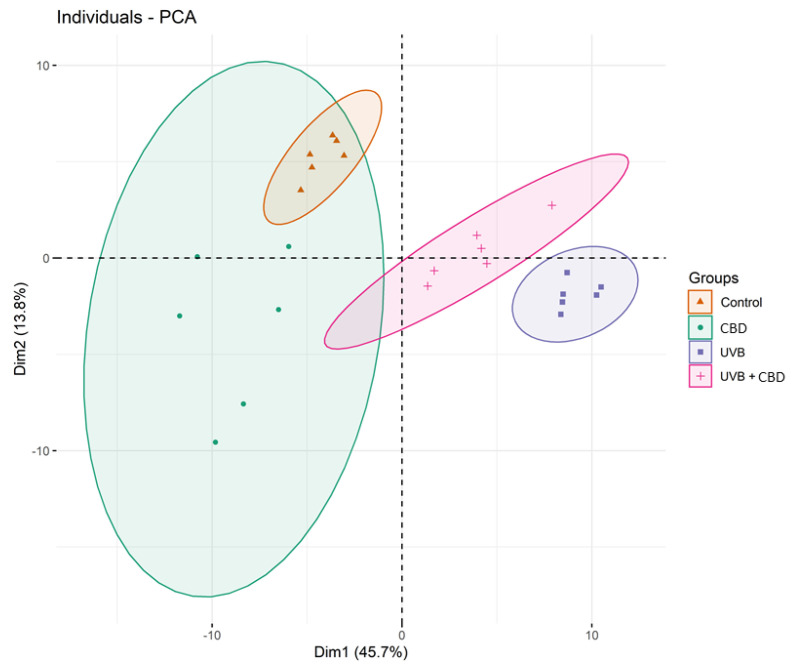
Principal component analysis (PCA) in a two-dimensional score plot of the relative abundance of phospholipid species in keratinocytes, isolated from the skin of healthy subjects (Control). These cells were not treated or treated with CBD (4 μM) or/and UVB (60 mJ/cm^2^). The following groups of keratinocytes were studied: Control, CBD, UVB, and UVB + CBD. All samples were analyzed after 24 h of treatment.

We then introduced UVB as the second independent variable in our study. The PCA model of the second data set of four groups of healthy not treated keratinocytes and treated with CBD (4 μM) or/and UVB (60 mJ/cm^2^) (Control; CBD; UVB; UVB + CBD) captured 59.5% of the total variance of the data, including principal component 1 (45.7%) and principal component 2 (13.8%) (Figure 3). The variation between keratinocytes groups was most pronounced along the first principal component, which was the major discriminating component and probably associated with UVB irradiation. The PCA model showed that the Control and CBD groups were scattered on the left region of the plot, while the UVB and UVB + CBD groups were scattered on the right region of the plot (Figure 3).

**Figure 4:**
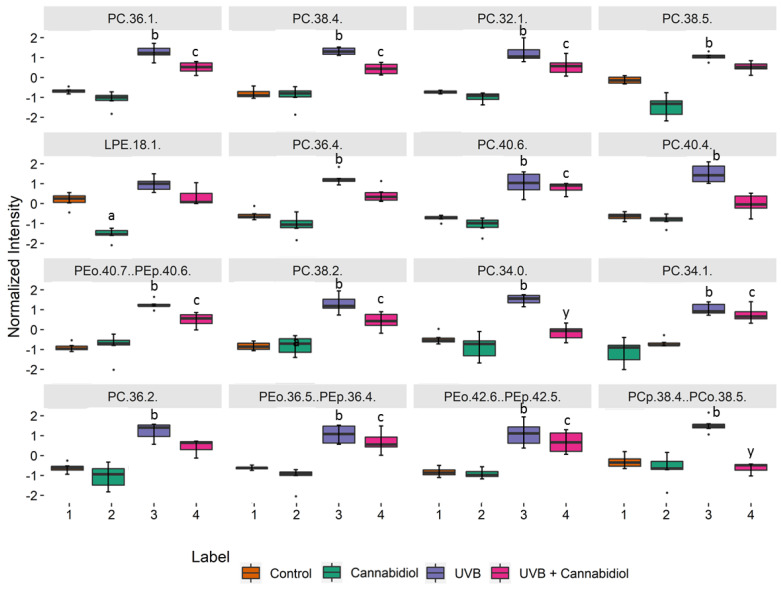
Boxplots of the 16 most discriminating PL molecular species (according to Kruskal–Wallis and the post hoc Dunn’s multiple comparisons tests) identified in keratinocytes isolated from the skin of healthy subjects (control). These cells were not treated or treated with CBD (4 μM) or/and UVB (60 mJ/cm^2^). The following groups of keratinocytes were examined: Control, CBD, UVB, and UVB + CBD; *p* < 0.05 was considered statistically significant: a, control vs. CBD; b, control vs. UVB; c − control vs. UVB + CBD; y − UVB vs. UVB + CBD.

We performed a univariate analysis (Kruskal–Wallis and the post hoc Dunn multiple comparison tests) in order to assess the variation of the relative abundance of the phospholipid species from the second set of data (Control; CBD; UVB; UVB + CBD), by comparing the keratinocytes exposed to UVB and treated with CBD to control keratinocytes and the keratinocytes treated with CBD only (Figure 4). Figure 4 shows the 16 most discriminating PL molecular species selected using the Kruskal–Wallis univariate statistical analysis (Table S5). We found that the PC species (PC (36:1), PC (38:4), PC (32:1), PC (38:5), PC (36:4), PC (40:6), PC (40:4), and PC (38:2)), and some ether-linked PE (PEo(36:5) and PEo(42:6)) levels were increased. We also observed a significant decrease in LPE (18:1) in the keratinocytes treated with CBD in comparison to the control keratinocytes (Figure 4).

As before, we tested if the changes in relative abundance of the main 25 phospholipid species from the second dataset selected according to Kruskal–Wallis analysis (lowest *p*-values) allowed discrimination between groups and created a dendrogram with a two-dimensional hierarchical clustering (Figure S2). In addition, the primary split in the upper hierarchical dendrogram shows that the samples independently clustered into four major groups. Clustering of individual phospholipids shows that they cluster into three main groups.

### 2.3. Comparison of the Phospholipid Profile of Psoriatic Keratinocytes Untreated and Treated with CBD (4 μM) or/and UVB (60 mJ/cm^2^) (Ps vs. Ps + CBD vs. Ps + UVB vs. Ps + UVB + CBD)

**Figure 5:**
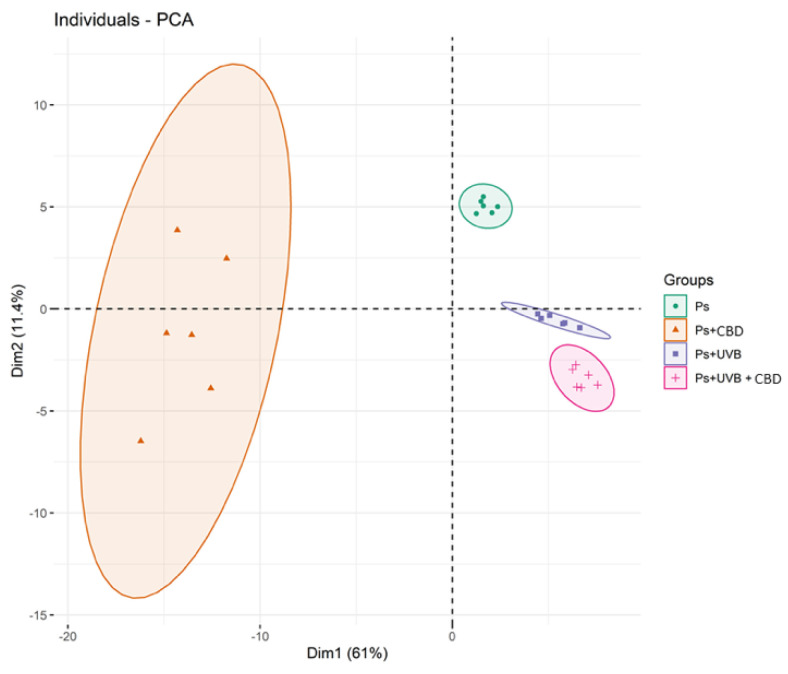
Principal component analysis (PCA) in a two-dimensional score plot of the relative abundance of phospholipid species in keratinocytes, isolated from the skin of psoriatic patients (Ps). These cells were not treated or treated with CBD (4 μM) or/and UVB (60 mJ/cm^2^). The following groups of keratinocytes were examined: Ps, Ps + CBD, Ps + UVB and Ps + UVB + CBD. All samples were analyzed after 24 h of treatment.

The PCA plots of the third and last data set covering four groups of not treated psoriatic keratinocytes or treated with CBD (4 μM) or/and UVB (60 mJ/cm^2^) (Ps; Ps + CBD; Ps + UVB; Ps + UVB + CBD) demonstrate that the subjects clustered into four distinct groups (Figure 5). The PCA model captured 72.4% of the total variance (PC1 (61%), PC2 (11.4%)), where the first principal component 1 was the main discriminating component. Three groups, Ps (psoriatic keratinocytes), Ps + UVB (psoriatic keratinocytes exposed to UVB), and Ps + UVB + CBD (psoriatic keratinocytes exposed to UVB and treated with CBD) were scattered on the right region of the PCA plot. Interestingly, psoriatic keratinocytes treated with CBD (and not UVB) patients were the only cluster that was scattered over the left region of the plot (Figure 5).

**Figure 6:**
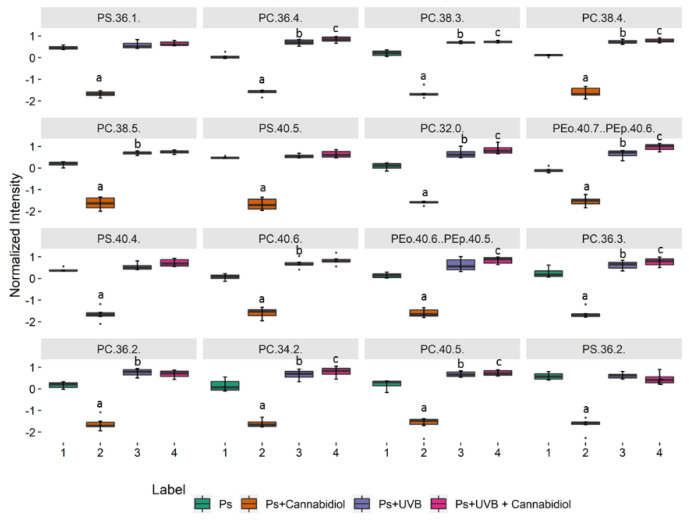
Boxplots of the 16 most discriminating PL molecular species (according to Kruskal–Wallis and post hoc Dunn’s multiple comparison tests) identified in keratinocytes isolated from the skin of psoriatic patients (Ps). These cells were not treated or treated with CBD (4 μM) or/and UVB (60 mJ/cm^2^). The following groups of keratinocytes were examined: Ps, Ps + CBD, Ps + UVB and Ps + UVB + CBD. *p* < 0.05 was considered statistically significant: a, Ps vs. Ps + CBD; b, Ps vs. Ps + UVB; c − Ps vs. Ps + UVB + CBD.

Figure 6 shows the 16 most discriminating PL molecular species selected by Kruskal–Wallis univariate statistical analysis (Supplementary Table S6) from the third data set (Ps; Ps + CBD; Ps + UVB; Ps + UVB + CBD). A significant decrease of the levels of PC species (PC (36:1), PC (36:4), PC (38:3), PC (38:4), PC (38:5), PC (32:0), PC (40:6), PC (36:3), PC (36:2), PC (34:2), and PC (40:5)), PS species (PS (40:5), PS (40:4) and PS (36:2) and two ether-linked PE (PEo (40:7) and PEo (40:6)), and a significant decrease of PI (36:1) were observed in psoriatic keratinocytes treated with CBD, compared to psoriatic keratinocytes as well as CBD-treated or not treated psoriatic keratinocytes after exposure to UVB (Figure 6).

Graphical presentations of changes observed in relative abundances of the main 25 phospholipid species from the third dataset selected using Kruskal–Wallis univariate analysis (lowest *p*-values) were represented in a dendrogram with a two-dimensional hierarchical clustering is shown in Figure S3. The primary split in the upper hierarchical dendrogram shows that the samples clustered independently into four main groups, but that not all samples of psoriatic keratinocyte exposed to UVB were properly clustered. The individual phospholipids were clustered into three main groups.

**Figure 7:**
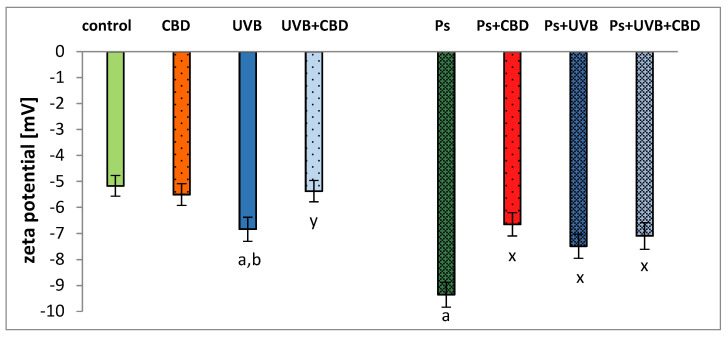
Zeta potential of keratinocytes isolated from the skin of healthy subjects (Control) and psoriatic patients (Ps). These cells were not treated and treated with CBD (4 μM) or/and UVB (60 mJ/cm^2^). All samples were analyzed after 24 h of treatment. Mean values ± SD are presented with statistically significant differences: (**a**) vs. control group; (**b**) vs. CBD treated group; (**x**) vs. psoriasis group; (**y**) vs. UVB treated group, *p* < 0.05.

Psoriatic keratinocytes showed an increase (81%) in negative zeta potential compared to control keratinocytes, probably due to changes in the composition of the membrane phospholipids (Figure 7). The CBD treatment significantly reduced the negative zeta potential. Interestingly, this effect was only observed for keratinocytes from patients with psoriasis. Following UVB irradiation, the negative zeta potential increased remarkably compared to the control keratinocytes. Control keratinocytes treated with CBD after UVB irradiation were characterized by a significantly reduced negative zeta potential (by up to 27%) compared to irradiated keratinocytes. The irradiation with UVB caused a decrease in the negative zeta potential of psoriatic keratinocytes (by 25%) compared to the psoriatic group. In contrast, treatment with CBD after UVB irradiation did not result in a statistically significant change in negative zeta potential comparison to psoriatic keratinocytes treated with CBD after UVB exposure.

## 3. Discussion

In addition to being the major structural component of cell membranes, phospholipids also play an important role in the regulation of cell metabolism [26,27]. PS and PE participate in cellular processes such as membrane fusion as well as autophagy and apoptosis [28]. However, PC, which is a substrate for phospholipase D, an enzyme that regulates keratinocyte differentiation, is required for metabolic processes in epidermal cells [29]. Altered phospholipid metabolism is involved in the pathogenesis of many diseases in humans, including psoriasis [5,6,30]. Despite this, there are limited studies on the phospholipid profiles of epidermal cells, particularly concerning changes in individual classes of phospholipids caused by pharmacotherapy or UVB phototherapy [31,32]. Since UV radiation is widely used in the treatment of skin diseases, and CBD has been proposed as a potential therapeutic compound, we examined the effect of both factors on the phospholipid profile of keratinocytes from healthy subjects and patients with psoriasis.

The results of this study show that the levels of several species of different classes of phospholipids, including phosphatidylcholines (PC), phosphatidylinositols (PI), phosphatidylserines (PS), and ether-linked PE (PEo), are reduced in the keratinocytes of patients with psoriasis (Figure 2, Table S4 and Figure S1). Previously, a reduction in phospholipid levels in plasma and mononuclear cells of patients with psoriasis has been observed [6,11]. PC species are involved in the differentiation of keratinocytes; therefore, a decrease in PC levels suggests a defect in this process, which results in the formation of a psoriatic lesion. The decrease in PC content may be due to the increased activity of lecithin-cholesterol acyltransferase (LCAT), which transfers fatty acids from PC to cholesterol [33]. Plasma in psoriasis patients has been shown to have reduced activity of haptoglobin, an acute phase glycoprotein, and an LCAT inhibitor [34,35].

Another possible mechanism leading to a reduction in PC levels may be associated with the transfer of acyl chains from PC to SM, catalyzed by PC-SM transacylase, an enzyme also present in keratinocytes [36]. This possibility is illustrated by the increase in SM (d41:2) in psoriatic keratinocytes observed in this study (Supplementary Table S4). Since the degradation of sphingomyelins by sphingomyelinase is one of the main pathways for the formation of ceramides [37], the observed increase in the level of SM (d41:2) may be due to the reduction in the activity of this enzyme in psoriatic keratinocytes [38]. Although our previous study showed a tendency of some ceramide classes to increase [13], the vast majority of other keratinocyte studies conducted in patients with psoriasis, showed decreased levels of ceramides, consistent with the changes in relative SM content observed in this study [12,14,15]. In addition, since ceramides are involved in regulating the permeability of the skin barrier [39], the observed increase in SM content in the keratinocytes of patients with psoriasis may indicate a mechanism leading to a loss of trans epidermal water as a result of a reduction in the synthesis ceramide from SM. Moreover, reduced levels of ceramides can also indicate their increased metabolism, leading to the formation of glycosphingolipid [38]. In support of this notion, glycosphingolipids, among other sphingolipids, are responsible for the negative charge of the cell membrane; our results confirm an increase in the negative charge of the keratinocyte membranes (Figure 7). The negative charge on the surface of the cell membrane also depends on the presence of anionic phospholipid species, in particular PS on the external surface of the cell membrane [40,41,42]. The translocation of phospholipids in cell membranes is considered to be one of the most important markers of the initial phase of apoptosis [43]. Relocation of CL takes place first, resulting in loss of membrane potential, and then PS is translocated to the external cell membrane. Thus, higher values of the negative zeta potential (Figure 7) of psoriatic keratinocytes indicate a translocation of PS into the outer layer of the membrane. This is important for the recognition of apoptotic cells by macrophages and their subsequent removal by phagocytosis [44]. Phospholipid metabolism is also an essential regulator of apoptosis, a highly desirable event in psoriasis to prevent the hyperproliferation of keratinocytes [45]. The process of phospholipid transformation catalyzed by cyclooxygenase and lipoxygenase leads to the generation of D-series prostaglandins and J-series prostaglandins. These mediators, through various metabolic pathways, induce apoptosis of keratinocytes [46]. Moreover, ROS-dependent lipid peroxidation end products, namely 4-HNE and 4-HHE, also induce apoptosis through their involvement in the receptor pathway of apoptosis [45].

The redox imbalance in apoptotic cells promotes the oxidation of PS by ROS, the overproduction of which is observed during inflammatory processes [47]. In addition, increased ROS production and reduced antioxidant capacity lead to increased lipid peroxidation [16]. This increase may, to some extent, explain the reduction in PS and PI levels observed in keratinocytes of patients with psoriasis (Supplementary Table S4 and Figure S1). We also suggest that the reduction of PI species could be the result of their increased phosphorylation to phosphoinositides (e.g., PI(4)P and PI(4,5)P2) by phosphatidylinositol kinases with increased activity in the psoriatic epidermis [48,49,50]. In general, the changes observed in the phospholipid profile of keratinocytes in patients with psoriasis appear to be associated with metabolic changes in these cells, the consequence of which is water loss and the initiation of cell signaling that induces cell death.

One of the most commonly used therapies in psoriasis is UVB phototherapy [17]. However, UV radiation is one of the exogenous physical factors which, through the induction of oxidative stress, leads to changes in the cellular metabolism of phospholipids [51,52]. Our study shows that UVB irradiation causes upregulation of PC species, PC plasmalogens (PCp), and PEo species, all of which are downregulated in non-irradiated psoriatic keratinocytes (Figure 4, Table S5 and Figure S2). These observations are in line with previous reports [53,54], but, in our study, the changes were more pronounced in healthy people than in patients. One possible explanation for this may be a significant increase in lipid peroxidation in the keratinocytes of patients with psoriasis, which has recently been reported [16]. The significant upregulation of PC species in UVB irradiated cells observed in this study corresponds to an increase in the negative zeta potential (Figure 7). The increase in phospholipids relative abundance shown above is most likely associated with the response of keratinocytes to oxidative stress resulting from UVB radiation. For example, our study shows upregulation of PC (34:1) (Figure 4), which is a PPARα nuclear receptor ligand [55] that regulates the response to inflammation by activating genes encoding anti-inflammatory mediators [56]. Furthermore, increasing PE levels increases the viability of mammalian cells by promoting autophagy as part of a survival mechanism [57]. Although UV radiation is one of the factors causing a change in phospholipid metabolism, our results show that exposure of psoriatic keratinocytes to UV radiation induces adaptive mechanisms that shift the metabolism of phospholipids towards the metabolism of healthy people.

The development of psoriasis and its treatment with UVB radiation shifted the redox balance toward oxidative conditions, leading to changes in the metabolism of phospholipids. Therefore, it seems reasonable to explore the antioxidant activity of CBD as a potential adjunct to the treatment of psoriasis, as recently suggested [58]. Treatment of keratinocytes from psoriatic patients with CBD leads to a further significant reduction in the level of PC, PS, and most PEo species (Figure 6, Table S6 and Figure S3) in the continuation of the pro-apoptotic changes observed in these cells during the development of psoriasis (Figure 2, Table S4, Figure S1). These changes are consistent with our latest research, which has shown increased oxidative stress and inflammation in psoriatic keratinocytes treated with CBD [16]. However, regardless of the general trend towards a reduction in phospholipid levels, the relative content of PEo molecular species, namely PEo (36:1) and PEo (40:4), is significantly increased in psoriatic keratinocytes treated with CBD.

Ether phospholipids support many important functions and play a role in several metabolic processes [59]. They are precursors of inflammatory lipid mediators/modulators, which modulate cell metabolism, including inflammation such as platelet-activating factor or thromboxanes, prostaglandins, and leukotrienes [60]. In addition, due to their ability to remove oxygen radicals, ether phospholipids have antioxidant properties [61,62]. Treatment of psoriatic keratinocytes with CBD results in significant upregulation of SM (d42:2) (Table S6 and Figure S3). The observed changes, namely upregulation of SM and downregulation of PC, may be responsible for the reduction of the negative zeta potential (Figure 7) of the keratinocyte membrane because the association constant of the negatively charged groups with H^+^ SM ions has a value greater than the association constant of these groups in PC [63,64]. It can be suggested that the bi-directional changes observed in the level of different species of phospholipids are a response of psoriatic keratinocytes to treatment with CBD. This could, on the one hand, reflect the potential therapeutic effect of this phytocannabinoid in relation to the antioxidant activity, but, on the other hand, confirm the pro-apoptotic effect of CBD as described in the literature [11].

The effect of CBD on the phospholipid profile of irradiated keratinocytes was also examined. Interestingly, the effects we observed were more pronounced in the cells of healthy people than in psoriatic patients. Our findings indicate that the treatment with CBD partially prevents the upregulation of PC, PCp, and PEo observed in keratinocytes of healthy individuals exposed to UVB radiation (Figure 4, Table S5, Figure S2). However, treatment of UVB irradiated psoriatic keratinocytes with CBD results in a significant decrease in the level of SM (d42:2) (Table S6, Figure S3). This reduction probably leads to increased production of ceramides catalyzed by sphingomyelinase, which consequently prevents the loss of transepidermal water. In contrast, the increase in SM (d42:2) observed in psoriatic keratinocytes not exposed to UVB radiation (Table S6, Figure S3) may indicate a potential role of CBD in increasing epidermal water loss. This loss of water can lead to cell death, which is highly desirable for psoriatic keratinocytes. Therefore, we believe that the observed response of keratinocytes from psoriasis patients to CBD treatment (i.e., rapid cell death of psoriatic cells) is more useful when carried out in the absence of UVB phototherapy. Moreover, the results of the recent study in psoriatic patients showed that topical treatment with an ointment enriched in CBD significantly improved skin parameters, symptoms, and the PASI index score [65]. Since no irritant or allergic reactions have been documented during therapy, topical administration of CBD ointment may be suggested as a safe, non-invasive, and most effective treatment for psoriasis.

## 4. Materials and Methods

### 4.1. Reagents/Chemicals

The phospholipid internal standards were purchased from Avanti Polar Lipids, Inc. (Alabaster, AL, USA). All chemicals were purchased from Sigma-Aldrich Chemical Co. (St. Louis, MO, USA); all solvents were of LC-MS grade. Milli-Q water was used for all experiments, filtered through a 0.22 mm filter and obtained using a Milli-Q Millipore system (Advantage A10, Millipore Corporation, Billerica, MA, USA).

### 4.2. Collection of Skin Samples

Skin tissue was collected from six untreated patients with a diagnosis of psoriasis vulgaris (three men and three women; age range 28–57 years, average 42; randomly selected from a cohort of 30 patients). Samples were also collected from six healthy volunteers (sex- and age-matched individuals forming a control group; age range 24–56 years, average 41). Eligible patients were those with a diagnosis of plaque psoriasis for at least six months, with at least 10% of the total body surface area affected. The severity of psoriasis was assessed using the Psoriasis Area and Severity Index (PASI) score (median 17; range 10–25). None of the patients or healthy subjects had received topical, injectable, or oral medication in the four weeks preceding the study. Individuals with a history of any other disorder were excluded from the study. None of the participants were smokers or heavy drinkers. The study was approved by the Local Bioethics Committee of the Medical University of Bialystok (Poland), No. R-I-002/289/2017. Written informed consent was obtained from all patients.

Immediately after the biopsy, skin fragments were taken for histopathological examination (hematoxylin-eosin staining). The remaining sample was washed in PBS with 50 U/mL of penicillin and 50 μg/mL of streptomycin and incubated overnight at 4 °C in 1 mg/mL dispase to separate the epidermis from the dermis. After incubation, the epidermis was digested using Trypsin/EDTA for 20 min at 37 °C. The separated keratinocytes were washed and resuspended in the cell culture medium.

### 4.3. Cell Culture and Treatment

Keratinocytes isolated from the skin of healthy subjects and patients with psoriasis were cultured in Keratinocyte Serum-Free Medium (Gibco, Grand Island, NY, USA) containing fetal bovine serum (10%), epidermal growth factor EGF 1–53 (5 µg/L), 50 U/mL of penicillin, and 50 μg/mL pf streptomycin. The cells were cultured in a humidified atmosphere of 5% CO_2_ at 37 °C. When the cells (passage 3) reached 70% confluence, they were subjected to further treatment.

The keratinocytes were irradiated with UVB on ice, 15 cm from the six (6 W) lamps (312 nm) (Bio-Link Crosslinker BLX 312/365; Vilber Lourmat, Germany), corresponding to 4.08 mW/cm^2^. An exposure dose of 60 mJ/cm^2^ (corresponding to 70% cell viability as measured by the MTT assay [66]) was selected.

The CBD treatment was carried out by culturing cells for 24 h in a medium containing 4 µM of CBD (Sigma-Aldrich, City, MO, USA). This concentration of CBD did not change the morphology or the proliferation of keratinocytes [23,67] or the cell viability measured by the MTT assay [66].

The keratinocytes were divided into eight experimental groups of six samples each:

- Group 1 [Control]: Keratinocytes from the skin of healthy subjects cultured in standard medium.
- Group 2 [CBD]: Keratinocytes of the skin of healthy subjects cultured for 24 h in a medium containing 4 µM of CBD.
- Group 3 [UVB]: Keratinocytes of the skin of healthy subjects exposed to UVB radiation.
- Group 4 [UVB + CBD]: Keratinocytes of the skin of healthy subjects exposed to UVB radiation and then cultured for 24 h in a medium containing 4 µM CBD.
- Group 5 [Ps]: Keratinocytes of the skin of psoriatic patients cultured in standard medium.
- Group 6 [Ps + CBD]: Keratinocytes of the skin of psoriatic patients cultured for 24 h in a medium containing 4 µM CBD.
- Group 7 [Ps + UVB]: Keratinocytes of the skin of psoriatic patients exposed to UVB radiation.
- Group 8 [Ps + UVB + CBD]: Keratinocytes of the skin of psoriatic patients exposed to UVB irradiation and cultured for 24 h in a medium containing 4 µM CBD.

The keratinocytes of each group were washed with PBS, collected by scraping into cold PBS and centrifuged.

### 4.4. Lipidomic Analysis

#### 4.4.1. Lipid Extraction

The cell pellet was resuspended in 1 mL of ultra-pure water (Milli-Q H_2_O), and the total lipids were extracted using the Bligh and Dyer method [68]. Briefly, 3.75 mL of a chloroform/methanol 1:2 (*v*/*v*) mixture was added to the cell homogenate, vortexed, and incubated on ice for 30 min. Then, 1.25 mL of chloroform was added, followed by additional vortexing. Then, 1.25 mL of Milli-Q H_2_O was added and vortexed again. The samples were centrifuged at 1000× *g* for 5 min at room temperature to obtain a two-phase system: an aqueous upper phase and an organic lower phase. The lipid extract was recovered from the organic phase. Then, 1.88 mL of chloroform was added to the aqueous phase followed by a vortex and a new centrifugation step, ensuring the complete extraction of the lipid phase. The organic phase was dried under a stream of nitrogen. After drying, the total lipid extracts were resuspended in 300 μL of chloroform, transferred to an amber vial, dried under a stream of nitrogen, and stored at −20 °C until analysis.

#### 4.4.2. Quantification of Phospholipids by Measurement of Phosphorus

The measurement of phosphorus was carried out according to Bartlett and Lewis [69]. The phospholipid extracts were dissolved in 300 μL of dichloromethane and a volume of 10 μL was transferred to a glass tube and dried under a stream of nitrogen. Then, 125 μL of 70% perchloric acid was added to each tube and the samples were incubated at 180 °C for 1 h. After cooling to room temperature, 825 μL of Milli-Q water, 125 μL of ammonium molybdate (2.5 g/100 mL) and 125 μL of ascorbic acid (0.1 g/1 mL) were added to each sample. The samples were then incubated at 100 °C for 10 min, before being immediately cooled in a cold-water bath. The phosphate standards (0.1 to 2 μg of phosphorus) underwent the same experimental procedure as the samples, but without the heating block step. Absorbance was measured at 797 nm in a Multiskan GO 1.00.38 Microplate Spectrophotometer (Thermo Scientific, Hudson, NH, USA). The amount of phosphorus present in each sample was calculated by linear regression. The amount of total phospholipid was determined by multiplying the amount of phosphorus by 25.

#### 4.4.3. UPLC-ESI-MS and MS/MS Analysis

The phospholipids were separated by hydrophilic interaction liquid chromatography (HILIC-LC-MS) using an ultra high-performance liquid chromatography (UPLC) system (Agilent 1290; Agilent Technologies, Santa Clara, CA, USA) coupled with a quadrupole time-of-flight mass spectrometer (QTOF) (Agilent 6540; Agilent Technologies, Santa Clara, CA, USA). Two mobile phases: mobile phase A [acetonitrile:methanol:water 2:1:1 (*v*/*v*/*v*) with 1 mM ammonium acetate] and mobile phase B [acetonitrile:methanol 3:2 (*v*/*v*) with 1 mM ammonium acetate] were used as the solvent system. The gradient started with 0% of mobile phase A, increased linearly to 100% of A in 20 min, maintained for 15 min, and returned to initial conditions in 10 min. Five μL of each sample containing 25 μg of phospholipid extract, diluted in 10 μL of phospholipid standards mixture (PC(14:0/14:0), LPC(19:0), PE(14:0/14:0), CL(14:0/14:0/14:0/14:0); PI(16:0/16:0); PS(14:0/14:0)) and 90 μL of eluent B was introduced into the Ascentis^®^ Si column (15 cm × 1 mm, 3 μm, Sigma-Aldrich) with a flow rate of 40 μL min^−1^. ESI Agilent Dual AJS ESI conditions were as follows: electrospray voltage, −3000 V; capillary temperature, 250 °C; sheath gas flow, 13 L/min. Data was collected in profile mode at an acquisition rate of 3 spectra/s in the extended dynamic range mode (2 GHz). MS/MS experiments were performed in a data-dependent acquisition mode (DDA) with an isolation width of ~1.3 Da. The parent ion scan spectra were acquired in the range of *m*/*z* 100–1500, and the collision energy was fixed at 35 eV. The PC, LPC, and SM species were analyzed in the LC-MS spectra in the negative ion mode as acetate anions adducts [M + CH_3_COO]^−^, while PE, LPE, PS, PI, and CL species were analyzed as [M − H]^−^ ions. The data acquisition was carried out with Mass Hunter data software (version B0.8.0) (Agilent Technologies, Santa Clara, CA, USA). The relative abundances of each ion were calculated by normalizing the area of each peak to the peak area of an internal standard. The standard deviation values of the abundances of each internal standard were between 4 and 8%.

#### 4.4.4. Identification of Phospholipids

The identification of each phospholipid species was based on the characteristic retention times and the analysis of the MS/MS spectra. MS/MS data in the negative ion mode were used to identify the fatty acid carboxylate anions fragments RCOO^−^, which allowed to assign the fatty acyl chains esterified to the PL precursor. In addition, the fragment ion at *m*/*z* 241.01 was used to confirm the [M − H]^−^ PI class precursor ions, while the neutral loss of 87 Da, allowed for identifying the precursor ions belonging to the PS class.

#### 4.4.5. Data Treatment

Data were processed using the MZmine 2.30 software [70]. Processing included filtering, peak detection, peak alignment and integration, and assignment of each phospholipid molecular species.

#### 4.4.6. Statistical Analysis

The experimental results are presented as the average ± standard deviation. The datasets composed of the XIC areas obtained by the HILIC-MS and MS/MS analysis were autoscaled and analyzed statistically. Principal component analysis (PCA) was performed with the R package FactoMineR [71], and ellipses were drawn, assuming a multivariate normal distribution and a level of 0.95. Univariate statistical analysis was performed using the Kruskal−Wallis test (R built-in function) following a post hoc Dunn test (R package dunn.test [72]. A *p*-value < 0.05 was considered as an indicator of statistical significance. The heatmaps (Figures S1–S3 in Supplementary Materials) were created using the R package pheatmap [73] using “Euclidea” as the clustering distance and “ward.D” as the clustering method. Univariate and multivariate statistical analyses were performed using R version 3.5.1 [74]. All graphics and boxplots were created using the R package ggplot2 [75].

### 4.5. Physicochemical Properties of Keratinocyte Cell Membranes

#### 4.5.1. Analysis of the Zeta Potential

The keratinocytes were suspended in 0.9% NaCl and placed in a measuring vessel. The zeta potential of cell membranes was measured using a Zetasizer Nano ZS apparatus (Malvern Instruments, Malvern, UK).

#### 4.5.2. Statistical Analysis

The data are expressed as average ± SD (for *n* = 6). The data were analyzed using one way ANOVA with the Scheffe’s F test for multiple comparisons to determine the significance of the differences between groups. A *p*-value < 0.05 was considered significant. Statistical analyses were performed using SPSS software (IBM Japan v.20.0, Tokyo, Japan).

## 5. Conclusions

In conclusion, CBD can modulate the phospholipid metabolism in keratinocyte in vitro in two different ways. First, CBD can promote the continuation of the pro-apoptotic changes observed in psoriatic keratinocytes. Second, CBD can regulate transepidermal water loss and antioxidant defence of UVB-irradiated keratinocytes from psoriasis patients, which may also have therapeutic value. However, more research is needed to confirm and explain the mechanisms behind the observed changes.

## Supplementary Materials

Supplementary Materials can be found at https://www.mdpi.com/1422-0067/21/18/6592/s1.

## Abbreviations

4-HNE	4-hydroxy-2-onenal
ANOVA	Analysis of variance
CBD	Cannabidiol
ESI	Electrospray ionization
HILIC	Hydrophilic interaction liquid chromatography
HPLC	High performance liquid chromatography
LPC	Lysophosphatidylcholine
LPE	Lysophosphoethanolamine
MDA	Malondialdehyde
PASI	Psoriasis area and severity index
PCA	Principal component analysis
PC	Phosphatidylcholine
PE	Phosphatidylethanolamine
PEo	Ether-linked phosphoethanolamine
PI	Phosphatidylinositol
PS	Phosphatidylserines
PLA_2_	Phospholipase A_2_
PLS-DA	Partial least squares-discriminate analysis
PUFAs	Polyunsaturated fatty acids
QTOF	Quadrupole time of flight mass spectrometer
ROS	Reactive oxygen species
SM	Sphingomyelin
