# Supplementary material for: Cannabidiol-Mediated Changes to the Phospholipid Profile of UVB-Irradiated Keratinocytes from Psoriatic Patients
Source: Int J Mol Sci. 2020 Sep 9;21(18):6592. doi: 10.3390/ijms21186592 (PMC7555222; doi:10.3390/ijms21186592)
Supplement: Supplementary file 1 [file ijms-21-06592-s001.pdf]

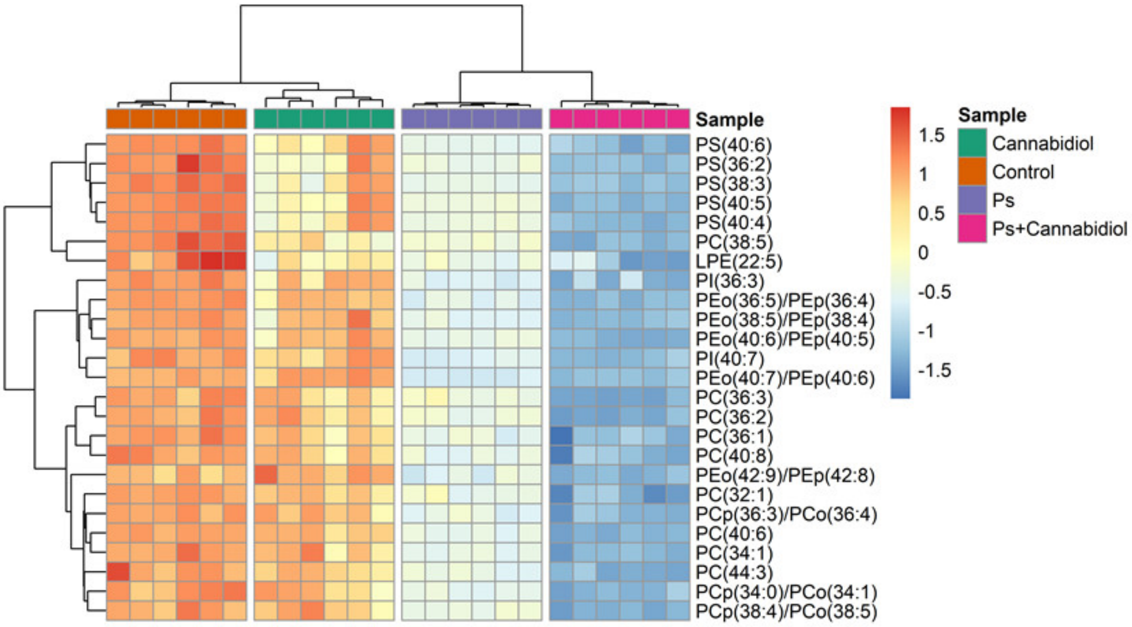


**Figure S1.** Two-dimensional hierarchical clustering heat map of the main 25 phospholipid species (lowest p-values in Kruskal–Wallis analysis) of the four groups (Control, CBD, Ps, and Ps+CBD). The relative abundance of each species is indicated on the colour scale, with the figures indicating the difference in fold compared to the overall average. The dendrogram at the top represents the clustering of the sample groups. The dendrogram on the left represents the clustering of individual phospholipid species (relative to the change in relative abundance).

The main 25 phospholipid species from the second dataset selected according to Kruskal–Wallis analysis (lowest p-values) are shown in Figure S2. The primary split in the upper hierarchical dendrogram shows that the samples independently clustered into four major groups. Clustering of individual phospholipids (with respect to the similarity of their changes in phospholipid expression) shows that they cluster into three main groups. The first group included two PC species and two PC plasmalogen species, which were most abundant in the UVB cluster. The second group contained two LPE species and one PC species, which were least abundant in the CBD groups and most abundant in the UVB group. The third group was mainly composed of PC species and ether-linked PE species which were more abundant in the UVB and UVB+CBD clusters.

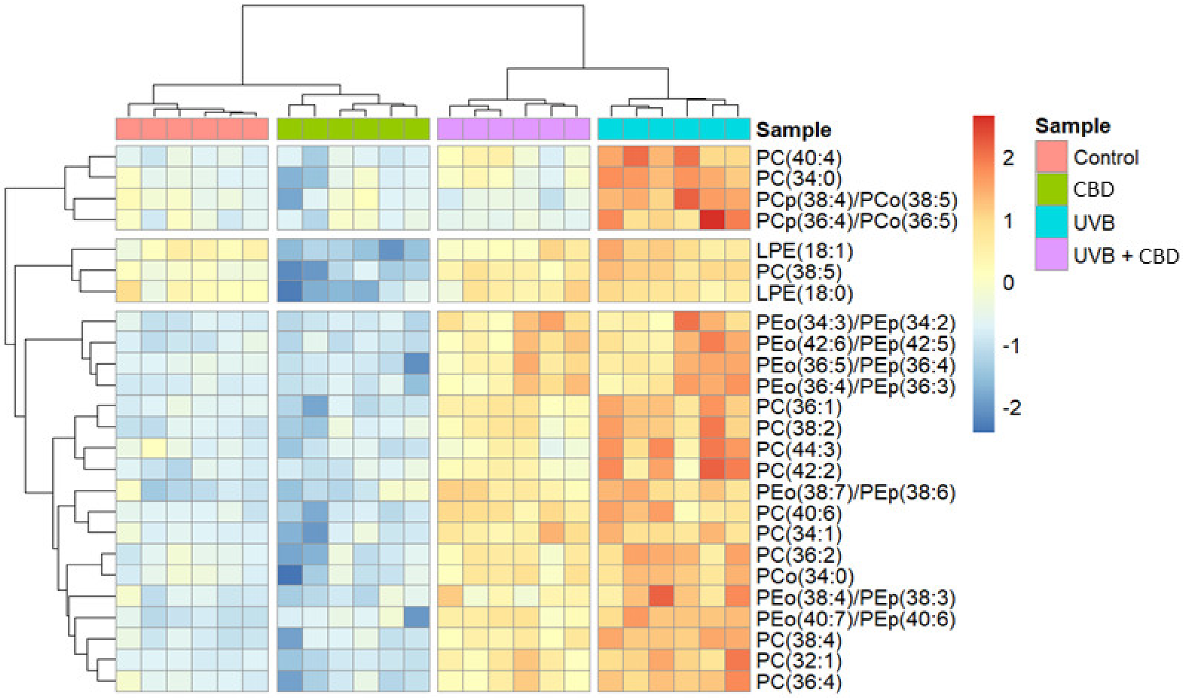


**Figure S2.** Two-dimensional hierarchical clustering heat map of the main 25 phospholipid species (lowest p-values in Kruskal–Wallis analysis) of the four groups (Control, CBD, UVB and UVB+CBD). The relative abundance of each species is shown on the colour scale, with the numbers indicating the fold difference from the overall mean. The dendrogram at the top represents the clustering of the sample groups. The dendrogram on the left represents the clustering of individual phospholipid species (relative to the observed changes in relative abundance).

Figure S3 shows the main 25 phospholipid species from the third dataset selected using Kruskal–Wallis univariate analysis (lowest p-values). The primary split in the upper hierarchical dendrogram shows that the samples clustered independently into four main groups, but that not all samples of psoriatic keratinocyte exposed to UVB were properly clustered. The individual phospholipids (grouped by similarity of changes of the phospholipid expression) were clustered into three main groups. The first was represented by the SM(d42:2) specie, which was most abundant in psoriatic keratinocytes treated with CBD and least abundant in psoriatic keratinocytes treated with CBD after UVB exposure. The second group consisted mainly of PS, while the third group included PC species and ether-linked PE species, which were less abundant in CBD-treated psoriatic keratinocytes compared to the other groups.

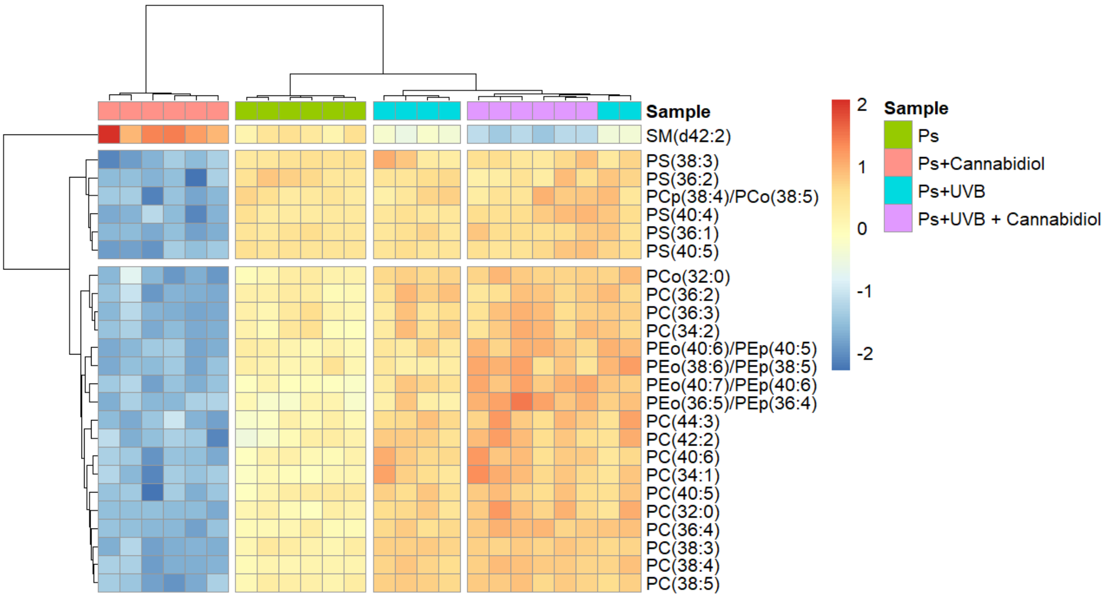


**Figure S3.** Two-dimensional hierarchical clustering heat map of the main 25 phospholipid species (according to lowest p-values in Kruskal–Wallis) from the four groups (Ps, Ps+CBD, Ps+UVB and Ps+UVB+CBD). The relative abundance of each species is shown on the colour scale, with the numbers indicating the fold difference from the overall average. The dendrogram at the top represents the clustering of the sample groups. The dendrogram at the left represents the clustering of individual phospholipid species (relative to the observed changes in relative abundance).

**Table S1**. Peak area of each phospholipid molecular species identified in the keratinocytes in keratinocytes, isolated from the skin of healthy subjects (Control) and psoriatic patients (Ps). These cells were not treated or treated with CBD (4µuM). The following groups of keratinocytes were examined: Control, CBD, Ps, and Ps+CBD. Data obtained using MZmine software (XLSX).

| | A | B | C | D | E | F | G | H | I | J | K | L | M | N | O | P | Q | R | S | T | U | V | W | X |
|---|---|---|---|---|---|---|---|---|---|---|---|---|---|---|---|---|---|---|---|---|---|---|---|---|
| 1 | Name | 505 | 506 | 507 | 508 | 509 | 510 | 505C | 506C | 507C | 508C | 509C | 510C | 602 | 603 | 605 | 606 | 607 | 611 | 602C | 603C | 605C | 606C | 607C |
| 2 | Label | Control | Control | Control | Control | Control | Control | Cannabidi | Cannabidi | Cannabidi | Cannabidi | Cannabidi | Cannabidi | Ps | Ps | Ps | Ps | Ps | Ps | Ps+Cannal | Ps+Cannal | Ps+Cannal | Ps+Cannal | Ps+Cannal |
| 3 | PC(34:1) | 1.446333 | 1.43833 | 1.629387 | 1.496522 | 1.446411 | 1.454116 | 1.403019 | 1.16507 | 1.451626 | 1.087743 | 1.580505 | 1.410492 | 1.745256 | 1.904952 | 1.875218 | 1.828324 | 1.915135 | 1.770435 | 1.118317 | 1.166173 | 0.895962 | 1.213026 | 1.161835 |
| 4 | PC(32:0) | 0.733743 | 0.746284 | 0.868121 | 0.832015 | 0.720831 | 0.773635 | 0.691998 | 0.48401 | 0.798734 | 0.664281 | 0.833396 | 0.74269 | 0.988753 | 1.076061 | 1.06516 | 0.955528 | 1.04578 | 0.902184 | 0.451975 | 0.450728 | 0.46171 | 0.464836 | 0.420711 |
| 5 | PC(38:4) | 0.879496 | 0.857457 | 0.993696 | 0.938606 | 0.874488 | 0.899067 | 0.856998 | 0.699431 | 0.919834 | 0.926577 | 0.973822 | 0.881086 | 1.150742 | 1.213143 | 1.215895 | 1.192196 | 1.214354 | 1.21645 | 0.518562 | 0.519463 | 0.478381 | 0.62223 | 0.537852 |
| 6 | PC(36:4) | 0.655584 | 0.634812 | 0.77488 | 0.68063 | 0.616991 | 0.640882 | 0.549122 | 0.43213 | 0.605761 | 0.525404 | 0.689643 | 0.570385 | 0.902728 | 0.919613 | 0.935729 | 0.949261 | 0.90723 | 1.056718 | 0.359348 | 0.40419 | 0.403651 | 0.423042 | 0.426976 |
| 7 | PC(36:1) | 0.4864 | 0.496075 | 0.477928 | 0.529477 | 0.501442 | 0.504524 | 0.461326 | 0.430119 | 0.487119 | 0.359572 | 0.439694 | 0.464162 | 0.607376 | 0.724794 | 0.655546 | 0.684463 | 0.703567 | 0.654693 | 0.492707 | 0.485385 | 0.313998 | 0.534638 | 0.479861 |
| 8 | PC(36:2) | 0.575008 | 0.581509 | 0.536709 | 0.630287 | 0.560395 | 0.591881 | 0.543583 | 0.432851 | 0.611378 | 0.451772 | 0.520607 | 0.574125 | 0.741972 | 0.66989 | 0.746439 | 0.782453 | 0.683148 | 0.771895 | 0.315026 | 0.320519 | 0.287352 | 0.350011 | 0.311599 |
| 9 | PC(34:2) | 0.538358 | 0.566329 | 0.388275 | 0.581082 | 0.551794 | 0.592944 | 0.529722 | 0.40906 | 0.557839 | 0.351108 | 0.372744 | 0.569226 | 0.654847 | 0.586596 | 0.610355 | 0.749997 | 0.57768 | 0.803729 | 0.2476 | 0.270988 | 0.249879 | 0.280645 | 0.252991 |
| 10 | PC(34:0) | 0.275808 | 0.263491 | 0.321146 | 0.286729 | 0.268907 | 0.277428 | 0.26084 | 0.206632 | 0.278127 | 0.216989 | 0.311512 | 0.269105 | 0.38746 | 0.363571 | 0.407206 | 0.367895 | 0.386026 | 0.353734 | 0.247226 | 0.265348 | 0.200747 | 0.255196 | 0.254269 |
| 11 | PC(38:5) | 0.344241 | 0.346573 | 0.388139 | 0.370446 | 0.355062 | 0.382913 | 0.255462 | 0.202372 | 0.272393 | 0.212515 | 0.305089 | 0.263556 | 0.406132 | 0.423609 | 0.454389 | 0.444767 | 0.453125 | 0.441783 | 0.198796 | 0.182536 | 0.193106 | 0.235108 | 0.237053 |
| 12 | PC(36:3) | 0.323107 | 0.306613 | 0.278044 | 0.336014 | 0.316562 | 0.332372 | 0.297568 | 0.231701 | 0.315854 | 0.226785 | 0.261362 | 0.31243 | 0.388577 | 0.374499 | 0.362646 | 0.424218 | 0.359073 | 0.476098 | 0.139479 | 0.144078 | 0.148796 | 0.151385 | 0.141721 |
| 13 | PCp(46:11 | 0.107828 | 0.106028 | 0.093263 | 0.102265 | 0.092972 | 0.103257 | 0.094831 | 0.093812 | 0.10431 | 0.111836 | 0.095128 | 0.105322 | 0.135378 | 0.114632 | 0.120851 | 0.125198 | 0.108129 | 0.128567 | 0.065529 | 0.093379 | 0.085563 | 0.078725 | 0.08434 |
| 14 | PC(38:3) | 0.201689 | 0.204953 | 0.22184 | 0.233516 | 0.217153 | 0.216781 | 0.223667 | 0.17072 | 0.240521 | 0.223856 | 0.228495 | 0.223285 | 0.245749 | 0.243019 | 0.273671 | 0.267576 | 0.264801 | 0.252463 | 0.124381 | 0.122029 | 0.115003 | 0.121471 | 0.121976 |
| 15 | PC(40:5) | 0.15052 | 0.145569 | 0.159852 | 0.157819 | 0.155316 | 0.152429 | 0.161529 | 0.138293 | 0.164131 | 0.128924 | 0.166246 | 0.158526 | 0.166577 | 0.204092 | 0.184067 | 0.198311 | 0.20334 | 0.202074 | 0.092895 | 0.104731 | 0.073595 | 0.100616 | 0.101875 |
| 16 | PCp(40:7), | 0.079895 | 0.075483 | 0.066719 | 0.07589 | 0.076415 | 0.082534 | 0.073358 | 0.058083 | 0.072855 | 0.078973 | 0.06405 | 0.079232 | 0.084162 | 0.094118 | 0.079517 | 0.091242 | 0.087078 | 0.09726 | 0.063841 | 0.06166 | 0.072825 | 0.060039 | 0.071732 |
| 17 | PC(40:6) | 0.136799 | 0.139597 | 0.138511 | 0.136191 | 0.12968 | 0.135233 | 0.125789 | 0.123076 | 0.132105 | 0.112229 | 0.134356 | 0.131176 | 0.14781 | 0.155856 | 0.165009 | 0.169448 | 0.165366 | 0.156189 | 0.085763 | 0.086388 | 0.072721 | 0.092809 | 0.078067 |
| 18 | PC(38:6) | 0.116199 | 0.115168 | 0.101505 | 0.130473 | 0.133423 | 0.131197 | 0.132089 | 0.108545 | 0.129168 | 0.083056 | 0.10049 | 0.129886 | 0.14099 | 0.127442 | 0.138978 | 0.141405 | 0.138151 | 0.13497 | 0.094507 | 0.07487 | 0.077817 | 0.099451 | 0.068384 |
| 19 | PCp(34:0), | 0.145329 | 0.126924 | 0.146323 | 0.149739 | 0.129915 | 0.152161 | 0.12212 | 0.09226 | 0.140755 | 0.119417 | 0.137544 | 0.143031 | 0.165698 | 0.158201 | 0.164587 | 0.170072 | 0.156158 | 0.180022 | 0.090222 | 0.091581 | 0.077126 | 0.091314 | 0.096229 |
| 20 | PC(32:1) | 0.08927 | 0.086795 | 0.090884 | 0.0902 | 0.088569 | 0.086257 | 0.08414 | 0.072444 | 0.08569 | 0.077467 | 0.08634 | 0.081945 | 0.119918 | 0.113186 | 0.120755 | 0.131045 | 0.117071 | 0.142189 | 0.061837 | 0.090185 | 0.059442 | 0.075449 | 0.091035 |
| 21 | PCp(36:3), | 0.115334 | 0.112587 | 0.120301 | 0.107727 | 0.114246 | 0.119703 | 0.110818 | 0.08236 | 0.104495 | 0.105071 | 0.116692 | 0.116112 | 0.118133 | 0.154226 | 0.122667 | 0.127271 | 0.141199 | 0.137723 | 0.070459 | 0.090195 | 0.050979 | 0.070854 | 0.084165 |
| 22 | PCp(42:8), | 0.083301 | 0.074634 | 0.080476 | 0.079113 | 0.078999 | 0.092897 | 0.077419 | 0.093862 | 0.07753 | 0.080549 | 0.078866 | 0.091039 | 0.088966 | 0.084471 | 0.090013 | 0.095709 | 0.089615 | 0.10364 | 0.094806 | 0.082889 | 0.079611 | 0.0878 | 0.086939 |
| 23 | PC(36:0) | 0.073262 | 0.064032 | 0.069906 | 0.06474 | 0.071013 | 0.071984 | 0.068883 | 0.069814 | 0.062798 | 0.054745 | 0.067808 | 0.069824 | 0.076322 | 0.082479 | 0.08169 | 0.095387 | 0.090268 | 0.099756 | 0.074803 | 0.096878 | 0.048802 | 0.096334 | 0.086847 |
| 24 | PCp(38:4), | 0.133818 | 0.129934 | 0.147941 | 0.138119 | 0.123595 | 0.125581 | 0.121123 | 0.095136 | 0.135357 | 0.122429 | 0.144982 | 0.123069 | 0.177194 | 0.162942 | 0.169571 | 0.156721 | 0.155053 | 0.158499 | 0.0689 | 0.077717 | 0.060751 | 0.081034 | 0.073833 |
| 25 | PCp(44:11 | 0.054699 | 0.057522 | 0.055774 | 0.065506 | 0.047818 | 0.058463 | 0.045906 | 0.046059 | 0.062885 | 0.064195 | 0.072743 | 0.056125 | 0.067284 | 0.075923 | 0.067505 | 0.068247 | 0.070214 | 0.067618 | 0.05304 | 0.077564 | 0.058403 | 0.045391 | 0.069739 |
| 26 | PCo(32:0) | 0.096005 | 0.096124 | 0.09853 | 0.095969 | 0.090329 | 0.093554 | 0.08762 | 0.055334 | 0.09309 | 0.060705 | 0.095574 | 0.090748 | 0.110797 | 0.123973 | 0.120171 | 0.1325 | 0.124158 | 0.135957 | 0.046878 | 0.041711 | 0.049549 | 0.04921 | 0.041827 |
| 27 | PC(40:4) | 0.08697 | 0.095112 | 0.093705 | 0.097715 | 0.089668 | 0.092094 | 0.087874 | 0.09138 | 0.09576 | 0.079615 | 0.09183 | 0.090252 | 0.129083 | 0.115637 | 0.134186 | 0.122424 | 0.121106 | 0.114288 | 0.072828 | 0.085981 | 0.053428 | 0.072636 | 0.08111 |
| 28 | PCp(38:3), | 0.083626 | 0.078372 | 0.089588 | 0.088162 | 0.080143 | 0.089089 | 0.077739 | 0.060651 | 0.085518 | 0.078659 | 0.086901 | 0.086416 | 0.093532 | 0.092842 | 0.097099 | 0.097313 | 0.093776 | 0.101018 | 0.056612 | 0.062054 | 0.038568 | 0.057796 | 0.065229 |
| 29 | PC(42:2) | 0.051352 | 0.054592 | 0.05485 | 0.0494 | 0.052964 | 0.055961 | 0.055083 | 0.05339 | 0.051376 | 0.050766 | 0.057044 | 0.058199 | 0.057816 | 0.077958 | 0.063087 | 0.069265 | 0.073662 | 0.076262 | 0.041662 | 0.043327 | 0.039832 | 0.046155 | 0.032549 |
| 30 | PC(44:3) | 0.064025 | 0.054859 | 0.056948 | 0.05723 | 0.051494 | 0.05271 | 0.048919 | 0.044774 | 0.054369 | 0.049965 | 0.054101 | 0.050074 | 0.066579 | 0.077316 | 0.067754 | 0.07681 | 0.072818 | 0.072872 | 0.043348 | 0.053338 | 0.046527 | 0.044449 | 0.041375 |
| 31 | PCp(46:12 | 0.04499 | 0.041973 | 0.043075 | 0.04415 | 0.041245 | 0.045861 | 0.042482 | 0.040096 | 0.045475 | 0.040074 | 0.044367 | 0.047237 | 0.049411 | 0.047797 | 0.04824 | 0.051269 | 0.046946 | 0.054504 | 0.030215 | 0.038299 | 0.041574 | 0.04078 | 0.034474 |
| 32 | PCp(36:4), | 0.042982 | 0.04293 | 0.049831 | 0.050532 | 0.044771 | 0.046957 | 0.043876 | 0.044159 | 0.049522 | 0.040597 | 0.048835 | 0.046018 | 0.049913 | 0.061254 | 0.054271 | 0.055282 | 0.056877 | 0.058414 | 0.037261 | 0.051076 | 0.03136 | 0.048618 | 0.04322 |
| 33 | PC(38:2) | 0.060487 | 0.064896 | 0.061423 | 0.067598 | 0.061937 | 0.066282 | 0.065653 | 0.057276 | 0.071654 | 0.055706 | 0.065109 | 0.070259 | 0.076043 | 0.092806 | 0.08201 | 0.084144 | 0.087104 | 0.082439 | 0.052757 | 0.067049 | 0.037559 | 0.061367 | 0.066895 |
| 34 | PC(40:7) | 0.075223 | 0.079225 | 0.061463 | 0.085262 | 0.073145 | 0.074968 | 0.074608 | 0.080956 | 0.086967 | 0.055609 | 0.062692 | 0.076467 | 0.080866 | 0.072838 | 0.094484 | 0.101901 | 0.084012 | 0.079859 | 0.057489 | 0.050789 | 0.026877 | 0.052814 | 0.050308 |
| 35 | PC(40:8) | 0.055317 | 0.054524 | 0.047158 | 0.051927 | 0.050795 | 0.051065 | 0.049271 | 0.043501 | 0.050369 | 0.035503 | 0.045743 | 0.049533 | 0.058424 | 0.05753 | 0.065104 | 0.068845 | 0.064648 | 0.056331 | 0.032803 | 0.045467 | 0.019886 | 0.038782 | 0.043124 |
| 36 | PCo(34:0) | 0.050124 | 0.050422 | 0.046306 | 0.053909 | 0.046713 | 0.052571 | 0.044378 | 0.030756 | 0.051214 | 0.040649 | 0.043991 | 0.049943 | 0.063728 | 0.059822 | 0.063209 | 0.066915 | 0.058481 | 0.067671 | 0.03758 | 0.041926 | 0.023967 | 0.040369 | 0.042818 |
| 37 | PCp(48:12 | 0.024244 | 0.026923 | 0.029277 | 0.026574 | 0.022584 | 0.027937 | 0.021003 | 0.018613 | 0.024714 | 0.020666 | 0.027228 | 0.025981 | 0.029005 | 0.030574 | 0.031045 | 0.030546 | 0.02863 | 0.034447 | 0.021154 | 0.030012 | 0.019605 | 0.022727 | 0.025498 |
| 38 | PCp(36:1), | 0.028751 | 0.030162 | 0.021531 | 0.028281 | 0.027046 | 0.027848 | 0.026235 | 0.017952 | 0.027433 | 0.021076 | 0.020885 | 0.027013 | 0.030556 | 0.032947 | 0.027335 | 0.032616 | 0.028715 | 0.033711 | 0.023144 | 0.018766 | 0.012771 | 0.016934 | 0.018304 |
| 39 | PC(30:0) | 0.03144 | 0.033311 | 0.03468 | 0.029004 | 0.033441 | 0.031247 | 0.031769 | 0.037256 | 0.027554 | 0.038669 | 0.042446 | 0.029684 | 0.033968 | 0.044194 | 0.037123 | 0.037479 | 0.046885 | 0.03833 | 0.023089 | 0.023466 | 0.019369 | 0.027787 | 0.037488 |
| 40 | LPC(16:0) | 0.082714 | 0.074519 | 0.111604 | 0.081843 | 0.088722 | 0.085082 | 0.021722 | 0.093158 | 0.086849 | 0.013993 | 0.117184 | 0.047444 | 0.086004 | 0.088078 | 0.106874 | 0.09687 | 0.097364 | 0.098288 | 0.151768 | 0.134098 | 0.377486 | 0.371303 | 0.151657 |
| 41 | LPC(18:0) | 0.07471 | 0.088416 | 0.111466 | 0.094356 | 0.091682 | 0.086074 | 0.046995 | 0.096266 | 0.078446 | 0.040552 | 0.117039 | 0.067776 | 0.09608 | 0.113184 | 0.11751 | 0.11141 | 0.109331 | 0.113442 | 0.184196 | 0.193809 | 0.398526 | 0.366257 | 0.192293 |

Table S1

**Table S2**. Peak area of each phospholipid molecular species identified in the keratinocytes, isolated from the skin of healthy subjects (Control). These cells were not treated or treated with CBD (4µuM) or/and UVB (60 mJ/cm$^2$). The following groups of keratinocytes were studied: Control, CBD, UVB, and UVB+CBD. Data obtained using MZmine software (XLSX).

| | A | B | C | D | E | F | G | H | I | J | K | L | M | N | O | P | Q | R | S | T | U |
|---|---|---|---|---|---|---|---|---|---|---|---|---|---|---|---|---|---|---|---|---|---|
| 1 | Name | 505 | 506 | 507 | 508 | 509 | 510 | 505C | 506C | 507C | 508C | 509C | 510C | 502B | 503B | 504B | 505B | 506B | 507B | 502BC | 503BC |
| 2 | Label | Control | Control | Control | Control | Control | Control | Cannabidi | Cannabidi | Cannabidi | Cannabidi | Cannabidi | Cannabidi | UVB | UVB | UVB | UVB | UVB | UVB | UVB + Can | UVB + |
| 3 | PC(34:1) | 1.4463326 | 1.4383297 | 1.6293867 | 1.4965215 | 1.4464114 | 1.4541158 | 1.4030191 | 1.1650698 | 1.4516259 | 1.0877429 | 1.5805051 | 1.4104923 | 2.0441794 | 2.3715745 | 2.1210499 | 2.1601267 | 2.3838580 | 2.0867539 | 1.9575307 | 2.0573 |
| 4 | PC(32:0) | 0.7337429 | 0.7462835 | 0.8681206 | 0.8320145 | 0.7208312 | 0.7736353 | 0.6919980 | 0.4840098 | 0.7987339 | 0.6642807 | 0.8333958 | 0.7426899 | 1.2437634 | 1.4058375 | 1.2621993 | 1.0790423 | 1.3707278 | 1.0307322 | 1.1277282 | 0.8740 |
| 5 | PC(38:4) | 0.8794958 | 0.8574571 | 0.9936959 | 0.9386060 | 0.8744880 | 0.8990669 | 0.8569982 | 0.6994306 | 0.9198339 | 0.9265766 | 0.9738220 | 0.8810855 | 1.4219880 | 1.5688296 | 1.4380947 | 1.4457855 | 1.5542199 | 1.5338339 | 1.2316317 | 1.1281 |
| 6 | PC(36:4) | 0.6555841 | 0.6348118 | 0.7748803 | 0.6806304 | 0.6169905 | 0.6408823 | 0.5491215 | 0.4321300 | 0.6057610 | 0.5254039 | 0.6896434 | 0.5703852 | 1.1498709 | 1.2044143 | 1.0965772 | 1.2178917 | 1.1974690 | 1.4725529 | 0.8341083 | 0.8621 |
| 7 | PC(36:1) | 0.4863999 | 0.4960750 | 0.4779283 | 0.5294767 | 0.5014415 | 0.5045237 | 0.4613262 | 0.4301194 | 0.4871185 | 0.3595722 | 0.4396940 | 0.4641618 | 0.7283514 | 0.9535139 | 0.8331633 | 0.8394484 | 0.9056933 | 0.8048631 | 0.6971509 | 0.6787 |
| 8 | PC(36:2) | 0.5750084 | 0.5815090 | 0.5367087 | 0.6302865 | 0.5603950 | 0.5918813 | 0.5435832 | 0.4328513 | 0.6113779 | 0.4517718 | 0.5206074 | 0.5741249 | 0.9089350 | 0.7582716 | 0.9561698 | 0.9346187 | 0.8059003 | 0.9519092 | 0.7864327 | 0.6939 |
| 9 | PC(34:2) | 0.5383583 | 0.5663290 | 0.3882748 | 0.5810818 | 0.5517941 | 0.5929439 | 0.5297224 | 0.4090603 | 0.5578385 | 0.3511084 | 0.3727438 | 0.5692261 | 0.7713365 | 0.6068627 | 0.8324346 | 0.9189130 | 0.6035654 | 1.0145147 | 0.7153868 | 0.6867 |
| 10 | PC(34:0) | 0.2758083 | 0.2634907 | 0.3211460 | 0.2867291 | 0.2689068 | 0.2774277 | 0.2608396 | 0.2066320 | 0.2781272 | 0.2169889 | 0.3115116 | 0.2691049 | 0.4991110 | 0.4636521 | 0.4932658 | 0.4490602 | 0.5031459 | 0.4300396 | 0.3480053 | 0.3201 |
| 11 | PC(38:5) | 0.3442411 | 0.3465730 | 0.3881393 | 0.3704457 | 0.3550616 | 0.3829126 | 0.2554615 | 0.2023715 | 0.2723926 | 0.2125149 | 0.3050887 | 0.2635563 | 0.4680222 | 0.5006458 | 0.5206392 | 0.5190881 | 0.5511889 | 0.5006528 | 0.4811680 | 0.4245 |
| 12 | PC(36:3) | 0.3231066 | 0.3066128 | 0.2780443 | 0.3360144 | 0.3165616 | 0.3323718 | 0.2975679 | 0.2317014 | 0.3158535 | 0.2267850 | 0.2613616 | 0.3124295 | 0.4540468 | 0.4423859 | 0.4472486 | 0.5124213 | 0.4015849 | 0.6198248 | 0.3725888 | 0.3660 |
| 13 | PCp(46:11 | 0.1078282 | 0.1060275 | 0.0932625 | 0.1022650 | 0.0929720 | 0.1032571 | 0.0948314 | 0.0938118 | 0.1043103 | 0.1118358 | 0.0951278 | 0.1053223 | 0.1629275 | 0.1232363 | 0.1484398 | 0.1481300 | 0.1232856 | 0.1538775 | 0.1092874 | 0.0933 |
| 14 | PC(38:3) | 0.2016886 | 0.2049527 | 0.2218400 | 0.2335155 | 0.2171528 | 0.2167813 | 0.2236674 | 0.1707195 | 0.2405209 | 0.2238561 | 0.2284952 | 0.2232848 | 0.2898101 | 0.2810857 | 0.3255019 | 0.3016374 | 0.3124483 | 0.2881450 | 0.2701746 | 0.2574 |
| 15 | PC(40:5) | 0.1505202 | 0.1455685 | 0.1598522 | 0.1578185 | 0.1553163 | 0.1524289 | 0.1615289 | 0.1382926 | 0.1641313 | 0.1289235 | 0.1662463 | 0.1585261 | 0.1826328 | 0.2626153 | 0.2082826 | 0.2388033 | 0.2513645 | 0.2517195 | 0.1694248 | 0.1780 |
| 16 | PCp(40:7), | 0.0798951 | 0.0754833 | 0.0667187 | 0.0758902 | 0.0764150 | 0.0825335 | 0.0733584 | 0.0580830 | 0.0728545 | 0.0789734 | 0.0640500 | 0.0792322 | 0.0884293 | 0.1127532 | 0.0923158 | 0.1065929 | 0.0977403 | 0.1119865 | 0.0881623 | 0.0799 |
| 17 | PC(40:6) | 0.1367994 | 0.1395970 | 0.1385109 | 0.1361910 | 0.1296797 | 0.1352327 | 0.1257893 | 0.1230763 | 0.1321053 | 0.1122289 | 0.1343556 | 0.1311757 | 0.1588208 | 0.1721153 | 0.1915064 | 0.2027039 | 0.2010514 | 0.1771452 | 0.1806168 | 0.1830 |
| 18 | PC(38:6) | 0.1161993 | 0.1151680 | 0.1015048 | 0.1304731 | 0.1334230 | 0.1311974 | 0.1320887 | 0.1085451 | 0.1291684 | 0.0830563 | 0.1004897 | 0.1298855 | 0.1657804 | 0.1397162 | 0.1764519 | 0.1523373 | 0.1428781 | 0.1387429 | 0.1704911 | 0.1442 |
| 19 | PCp(34:0), | 0.1453287 | 0.1269238 | 0.1463232 | 0.1497393 | 0.1299149 | 0.1521606 | 0.1221200 | 0.0922604 | 0.1407550 | 0.1194167 | 0.1375439 | 0.1430309 | 0.1860679 | 0.1894779 | 0.1828497 | 0.1904038 | 0.1824007 | 0.2078829 | 0.1661289 | 0.1335 |
| 20 | PC(32:1) | 0.0892697 | 0.0867947 | 0.0908838 | 0.0901996 | 0.0885686 | 0.0862574 | 0.0841402 | 0.0724443 | 0.0856896 | 0.0774670 | 0.0863396 | 0.0819445 | 0.1505659 | 0.1395765 | 0.1506261 | 0.1718906 | 0.1455741 | 0.1981211 | 0.1163612 | 0.1282 |
| 21 | PCp(36:3), | 0.1153342 | 0.1125872 | 0.1203013 | 0.1077265 | 0.1142456 | 0.1197028 | 0.1108182 | 0.0823599 | 0.1044947 | 0.1050709 | 0.1166923 | 0.1161118 | 0.1209307 | 0.1958639 | 0.1250317 | 0.1468159 | 0.1681524 | 0.1557423 | 0.1220011 | 0.1140 |
| 22 | PCp(42:8), | 0.0833005 | 0.0746339 | 0.0804759 | 0.0791127 | 0.0789987 | 0.0928967 | 0.0774187 | 0.0938615 | 0.0775304 | 0.0805489 | 0.0788664 | 0.0910387 | 0.0946313 | 0.0943075 | 0.0995491 | 0.1123057 | 0.1002312 | 0.1143834 | 0.1023814 | 0.0811 |
| 23 | PC(36:0) | 0.0732623 | 0.0640315 | 0.0699056 | 0.0647400 | 0.0710131 | 0.0719836 | 0.0688827 | 0.0698138 | 0.0627978 | 0.0547454 | 0.0678084 | 0.0698241 | 0.0793812 | 0.1009257 | 0.0934742 | 0.1260346 | 0.1095225 | 0.1275281 | 0.0875320 | 0.0890 |
| 24 | PCp(38:4), | 0.1338184 | 0.1299340 | 0.1479409 | 0.1381191 | 0.1235949 | 0.1255806 | 0.1211230 | 0.0951358 | 0.1353567 | 0.1224288 | 0.1449821 | 0.1230690 | 0.2205695 | 0.1959495 | 0.1912010 | 0.1753235 | 0.1865120 | 0.1914182 | 0.1267610 | 0.1183 |
| 25 | PCp(44:11 | 0.0546987 | 0.0575224 | 0.0557736 | 0.0655056 | 0.0478182 | 0.0584634 | 0.0459055 | 0.0460593 | 0.0628854 | 0.0641947 | 0.0727427 | 0.0561249 | 0.0798695 | 0.0943238 | 0.0792356 | 0.0709879 | 0.0926106 | 0.0767735 | 0.0632282 | 0.0492 |

Table S2_C-UVB-CBD-UVB+CBD

**Table S3**. Peak area of each phospholipid molecular species identified in the keratinocytes, isolated from the skin of psoriatic patients (Ps). These cells were not treated or treated with CBD (4µuM) or/and UVB (60 mJ/cm²). The following groups of keratinocytes were examined: Ps, Ps+CBD, Ps+UVB and Ps+UVB+CBD. Data obtained using MZmine software (XLSX).

| | A | B | C | D | E | F | G | H | I | J | K | L | M | N | O | P | Q | R | S | T | U |
|---|---|---|---|---|---|---|---|---|---|---|---|---|---|---|---|---|---|---|---|---|---|
| 1 | Name | 602 | 603 | 605 | 606 | 607 | 611 | 602C | 603C | 605C | 606C | 607C | 611C | 602B | 603B | 604B | 605B | 606B | 607B | 602BC | 603BC |
| 2 | Label | Ps | Ps | Ps | Ps | Ps | Ps | Ps+Cannal | Ps+Cannal | Ps+Cannal | Ps+Cannal | Ps+Cannal | Ps+Cannal | Ps+UVB | Ps+UVB | Ps+UVB | Ps+UVB | Ps+UVB | Ps+UVB | Ps+UVB + | Ps+UV |
| 3 | PC(34:1) | 1.7452560 | 1.9049521 | 1.8752183 | 1.8283241 | 1.9151347 | 1.7704348 | 1.1183167 | 1.1661726 | 0.8959620 | 1.2130262 | 1.1618350 | 1.0554332 | 2.1721456 | 2.4676750 | 2.2862662 | 2.1594336 | 2.7240876 | 2.3833594 | 2.3241958 | 2.6404 |
| 4 | PC(32:0) | 0.9887531 | 1.0760605 | 1.0651600 | 0.9555284 | 1.0457795 | 0.9021837 | 0.4519750 | 0.4507276 | 0.4617099 | 0.4648363 | 0.4207106 | 0.4595323 | 1.2117170 | 1.5546922 | 1.4015375 | 1.2079790 | 1.2681122 | 1.3151133 | 1.3207715 | 1.6946 |
| 5 | PC(38:4) | 1.1507419 | 1.2131433 | 1.2158953 | 1.1921958 | 1.2143539 | 1.2164504 | 0.5185622 | 0.5194633 | 0.4783809 | 0.6222299 | 0.5378520 | 0.6236049 | 1.5764013 | 1.6941547 | 1.5847979 | 1.5221956 | 1.5666380 | 1.6664154 | 1.6236934 | 1.7449 |
| 6 | PC(36:4) | 0.9027275 | 0.9196131 | 0.9357287 | 0.9492610 | 0.9072297 | 1.0567176 | 0.3593482 | 0.4041896 | 0.4036513 | 0.4230422 | 0.4269760 | 0.4201057 | 1.3975124 | 1.4110543 | 1.2050315 | 1.2744779 | 1.2677628 | 1.3753179 | 1.4953383 | 1.5098 |
| 7 | PC(36:1) | 0.6073756 | 0.7247944 | 0.6555458 | 0.6844625 | 0.7035674 | 0.6546934 | 0.4927072 | 0.4853851 | 0.3139976 | 0.5346381 | 0.4798608 | 0.4272402 | 0.8735276 | 1.0832778 | 1.0352380 | 0.8975357 | 0.9523489 | 0.9046554 | 0.8211160 | 1.0182 |
| 8 | PC(36:2) | 0.7419717 | 0.6698903 | 0.7464392 | 0.7824526 | 0.6831477 | 0.7718952 | 0.3150255 | 0.3205188 | 0.2873517 | 0.3500106 | 0.3115993 | 0.4195542 | 1.0050007 | 0.8900833 | 0.9340194 | 0.9891766 | 0.8460101 | 1.0256172 | 0.9748507 | 0.8633 |
| 9 | PC(34:2) | 0.6548474 | 0.5865958 | 0.6103547 | 0.7499974 | 0.5776798 | 0.8037293 | 0.2475999 | 0.2709878 | 0.2498785 | 0.2806450 | 0.2529912 | 0.3121268 | 0.9314550 | 0.8418237 | 0.7906535 | 0.8953469 | 0.7189803 | 0.9696994 | 0.9966568 | 0.9007 |
| 10 | PC(34:0) | 0.3874597 | 0.3635714 | 0.4072059 | 0.3678946 | 0.3860263 | 0.3537337 | 0.2472256 | 0.2653475 | 0.2007472 | 0.2551961 | 0.2542689 | 0.2265834 | 0.5257053 | 0.4467565 | 0.5316480 | 0.5274137 | 0.4595258 | 0.4437235 | 0.5151912 | 0.4378 |
| 11 | PC(38:5) | 0.4061316 | 0.4236094 | 0.4543893 | 0.4447669 | 0.4531253 | 0.4417827 | 0.1987959 | 0.1825360 | 0.1931064 | 0.2351084 | 0.2370528 | 0.2256816 | 0.5103450 | 0.5426437 | 0.5377243 | 0.5209110 | 0.5417566 | 0.5551441 | 0.5205519 | 0.5534 |
| 12 | PC(36:3) | 0.3885767 | 0.3744994 | 0.3626464 | 0.4242179 | 0.3590733 | 0.4760983 | 0.1394789 | 0.1440784 | 0.1487956 | 0.1513852 | 0.1417206 | 0.1891185 | 0.5135430 | 0.4993575 | 0.4364813 | 0.4705424 | 0.4152323 | 0.5337062 | 0.5546264 | 0.5393 |
| 13 | PCp(46:11 | 0.1353778 | 0.1146319 | 0.1208511 | 0.1251975 | 0.1081288 | 0.1285673 | 0.0655287 | 0.0933793 | 0.0855634 | 0.0787248 | 0.0843403 | 0.0752803 | 0.1549805 | 0.1188504 | 0.1331296 | 0.1514389 | 0.1086046 | 0.1360756 | 0.1518809 | 0.1164 |
| 14 | PC(38:3) | 0.2457493 | 0.2430192 | 0.2736709 | 0.2675764 | 0.2648006 | 0.2524632 | 0.1243809 | 0.1220286 | 0.1150034 | 0.1214709 | 0.1219760 | 0.1461629 | 0.3062774 | 0.3087559 | 0.3212076 | 0.3108168 | 0.3140005 | 0.3146449 | 0.3096465 | 0.3121 |
| 15 | PC(40:5) | 0.1665765 | 0.2040919 | 0.1840674 | 0.1983109 | 0.2033404 | 0.2020742 | 0.0928946 | 0.1047305 | 0.0735948 | 0.1006157 | 0.1018749 | 0.1041793 | 0.2202308 | 0.2435632 | 0.2412019 | 0.2181024 | 0.2331501 | 0.2246055 | 0.2246354 | 0.2484 |
| 16 | PCp(40:7), | 0.0841622 | 0.0941183 | 0.0795173 | 0.0912415 | 0.0870777 | 0.0972600 | 0.0638411 | 0.0616602 | 0.0728245 | 0.0600388 | 0.0717320 | 0.0615733 | 0.0935716 | 0.1087443 | 0.0970747 | 0.0924915 | 0.0911181 | 0.1038932 | 0.0963787 | 0.1120 |
| 17 | PC(40:6) | 0.1478101 | 0.1558561 | 0.1650086 | 0.1694475 | 0.1653655 | 0.1561889 | 0.0857625 | 0.0863882 | 0.0727205 | 0.0928085 | 0.0780668 | 0.0896287 | 0.1825159 | 0.2003063 | 0.1995945 | 0.1985586 | 0.2333639 | 0.2086060 | 0.1934669 | 0.2123 |
| 18 | PC(38:6) | 0.1409898 | 0.1274421 | 0.1389783 | 0.1414052 | 0.1381505 | 0.1349702 | 0.0945073 | 0.0748695 | 0.0778173 | 0.0994514 | 0.0683840 | 0.0753420 | 0.1540314 | 0.1641454 | 0.1630216 | 0.1593637 | 0.1493407 | 0.1753937 | 0.1617330 | 0.1723 |
| 19 | PCp(34:0), | 0.1656983 | 0.1582008 | 0.1645865 | 0.1700716 | 0.1561578 | 0.1800217 | 0.0902218 | 0.0915814 | 0.0771259 | 0.0913140 | 0.0962288 | 0.1153902 | 0.1809094 | 0.1760775 | 0.1709724 | 0.1730648 | 0.1605614 | 0.1965477 | 0.1917639 | 0.1866 |
| 20 | PC(32:1) | 0.1199178 | 0.1131856 | 0.1207550 | 0.1310451 | 0.1170714 | 0.1421892 | 0.0618367 | 0.0901850 | 0.0594423 | 0.0754488 | 0.0910348 | 0.0691968 | 0.1924201 | 0.1800104 | 0.1611838 | 0.1823624 | 0.1695662 | 0.1918133 | 0.1962685 | 0.1836 |
| 21 | PCp(36:3), | 0.1181325 | 0.1542256 | 0.1226665 | 0.1272712 | 0.1411990 | 0.1377226 | 0.0704586 | 0.0901950 | 0.0509789 | 0.0708541 | 0.0841649 | 0.0698835 | 0.1253149 | 0.1700183 | 0.1428820 | 0.1230967 | 0.1409731 | 0.1403668 | 0.1303275 | 0.1768 |
| 22 | PCp(42:8), | 0.0889659 | 0.0844707 | 0.0900125 | 0.0957092 | 0.0896149 | 0.1036400 | 0.0948058 | 0.0828893 | 0.0796107 | 0.0878002 | 0.0869392 | 0.0741143 | 0.0999532 | 0.0930952 | 0.0934240 | 0.1003607 | 0.0920524 | 0.1142217 | 0.0969546 | 0.0903 |
| 23 | PC(36:0) | 0.0763217 | 0.0824786 | 0.0816899 | 0.0953873 | 0.0902678 | 0.0997558 | 0.0748034 | 0.0968783 | 0.0488019 | 0.0963341 | 0.0868470 | 0.0674638 | 0.1094149 | 0.1192971 | 0.1066543 | 0.1147700 | 0.1132320 | 0.1213031 | 0.1072266 | 0.1169 |
| 24 | PCp(38:4), | 0.1771940 | 0.1629418 | 0.1695709 | 0.1567213 | 0.1550534 | 0.1584994 | 0.0689001 | 0.0777165 | 0.0607510 | 0.0810338 | 0.0738330 | 0.0814653 | 0.1934604 | 0.1611168 | 0.1781512 | 0.1807937 | 0.1485257 | 0.1599893 | 0.1992642 | 0.1659 |
| 25 | PCp(44:11 | 0.0672841 | 0.0759231 | 0.0675046 | 0.0682468 | 0.0702144 | 0.0676184 | 0.0530396 | 0.0775637 | 0.0584026 | 0.0453913 | 0.0697386 | 0.0625708 | 0.0727677 | 0.0837053 | 0.0687535 | 0.0687928 | 0.0722506 | 0.0731294 | 0.0764061 | 0.0878 |

Tbale S3_Ps-Ps+UVB-Ps+CBD-Ps+UV

We used the results of the univariate analysis to create a dendrogram with a two-dimensional hierarchical clustering, using the 25 main phospholipid species, according to Kruskal–Wallis analysis (Figure S1). The primary split in the upper hierarchical dendrogram shows that the samples are clustered independently in the four main experimental groups. The clustering of the individual phospholipids (with regard to their similar expression changes) shows that they cluster into one main group mainly composed of PC, PS, PI and ether-linked PE species which were more abundant in the Control and CBD cluster. All the phospholipid species were least abundant in Ps+CBD group.

**Table S4.** The alteration observed in the molecular species of PC, PEo. PS, PI and SM in the keratinocytes, isolated from the skin of healthy subjects (Control) and psoriatic patients (Ps) comparing control with CBD, control with Ps, control with Ps+CBD, Ps with Ps+CBD along with their respective fold change. All the alteration are significant at the $P < 0.05$ level. CBD, 4µuM.

| Phospholipid specie | CBD vs control | | Ps vs control | | Ps+CBD vs control | | Ps+CBD vs Ps | |
|---|---|---|---|---|---|---|---|---|
| | Adaptation | Fold change | Adaptation | Fold change | Adaptation | Fold change | Adaptation | Fold change |
| PC(32:1) | | | ↓ | 1.53 | ↓ | 0.71 | ↓ | 0.60 |
| PC (36:1) | | | ↓ | 1.59 | ↓ | 0.72 | ↓ | 0.68 |
| PC (40:6) | | | ↓ | 1.82 | ↓ | 0.59 | ↓ | 0.53 |
| PC (40:8) | | | ↓ | 1.79 | ↓ | 0.63 | ↓ | 0.57 |
| PC (38:5) | | | ↓ | 1.78 | ↓ | 0.81 | ↓ | 0.48 |
| PC (44:3) | | | ↓ | 1.66 | ↓ | 0.67 | ↓ | 0.62 |
| PC (36:3) | | | ↓ | 1.70 | ↓ | 0.68 | ↓ | 0.38 |
| PI (36:3) | | | ↓ | 2.33 | ↓ | 0.51 | | |
| PS (40:5) | | | ↓ | 2.06 | ↓ | 0.67 | ↓ | 0.36 |
| PS (36:2) | | | ↓ | 2.17 | ↓ | 0.68 | ↓ | 0.42 |
| PS (40:6) | | | ↓ | 2.07 | ↓ | 0.60 | ↓ | 0.53 |
| PS (38:3) | | | ↓ | 2.01 | ↓ | 0.66 | ↓ | 0.55 |
| PS (40:4) | | | ↓ | 2.01 | ↓ | 0.68 | ↓ | 0.43 |
| SM(d41:2) | | | ↑ | 0.95 | ↑ | 0.79 | | |
| PEo(36:5)/PEp(36:4) | | | ↓ | 1.77 | ↓ | 0.63 | ↓ | 0.67 |
| PEo(38:5)/PEp(38:4) | | | ↓ | 1.82 | ↓ | 0.60 | ↓ | 0.64 |
| PEo(36:1)/PEp(36:0) | | | | | ↓ | 1.61 | ↑ | 2.75 |
| PEo(40:4)/PEp(40:3) | | | | | ↓ | 0.60 | ↑ | 1.16 |
| PCp(34:0)/PCo(34:1) | | | ↓ | 1.83 | ↓ | 0.61 | ↓ | 0.56 |

**Table S5.** The alteration observed in the molecular species of PC, PEo, PCp and LPE in the keratinocytes, isolated from the skin of healthy subjects (Control) comparing control with CBD, control with UVB, control with UVB+CBD, UVB with UVB+CBD along with their respective fold change. All the alteration are significant at the $P < 0.05$ level. CBD, 4μ~~u~~M; UVB, (60 mJ/$cm^2$).

| **Phospholipid specie** | **CBD vs control** | | **UVB vs control** | | **UVB+CBD vs control** | | **UVB+CBD vs UVB** | |
|---|---|---|---|---|---|---|---|---|
| | **Adaptation** | **Fold change** | **Adaptation** | **Fold change** | **Adaptation** | **Fold change** | **Adaptation** | **Fold change** |
| PC(36:1) | | | ↑ | 1.69 | ↑ | 0.73 | | |
| PC (38:4) | | | ↑ | 1.64 | ↑ | 0.75 | | |
| PC (32:1) | | | ↑ | 1.79 | ↑ | 0.68 | | |
| PC (38:5) | | | ↑ | 1.41 | | | | |
| PC (36:4) | | | ↑ | 1.82 | | | | |
| PC (40:6) | | | ↑ | 1.35 | ↑ | 0.77 | | |
| PC (40:4) | | | ↑ | 1.65 | | | ↓ | 0.70 |
| PC (38:2) | | | ↑ | 1.65 | ↑ | 0.74 | | |
| PC (34:0) | | | ↑ | 1.67 | | | ↓ | 0.65 |
| PC (34:1) | | | ↑ | 1.47 | ↑ | 0.72 | | |
| PC (36:2) | | | ↑ | 1.54 | | | | |
| PEo(36:5)/PEp(36:4) | | | ↑ | 1.43 | ↑ | 0.76 | | |
| PEo(42:6)/PEp(42:5) | | | ↑ | 1.48 | ↑ | 0.73 | | |
| PEo(40:7)/PEp(40:6) | | | | | | | | |
| PCp(38:4)/PCo(38:5) | | | ↑ | 1.44 | | | ↓ | 1.56 |
| LPE (18:1) | ↓ | 0.71 | | | | | | |

**Table S6.** The alteration observed in the molecular species of PC, PS, PEo and SM in the keratinocytes, isolated from the skin of psoriatic patients (Ps) comparing Ps with Ps+CBD, Ps with Ps+UVB, Ps with Ps+UVB+CBD, Ps+UVB+CBD vs PS+UVB along with their respective fold change. All the alteration are significant at the P < 0.05 level. CBD, 4µuM; UVB, (60 mJ/cm[2]).

| **Phospholipid specie** | **Ps+CBD vs Ps** | | **Ps+UVB vs Ps** | | **Ps+UVB+CBD vs Ps** | | **Ps+UVB+CBD vs Ps+UVB** | |
|---|---|---|---|---|---|---|---|---|
| | **Adaptation** | **Fold change** | **Adaptation** | **Fold change** | **Adaptation** | **Fold change** | **Adaptation** | **Fold change** |
| PC(36:1) | ↓ | 0.68 | | | | | | |
| PC (36:4) | ↓ | 0.43 | ↑ | 1.40 | ↑ | 1.50 | | |
| PC (38:3) | ↓ | 0.49 | ↑ | 1.21 | ↑ | 1.22 | | |
| PC (38:4) | ↓ | 0.46 | ↑ | 1.33 | ↑ | 1.37 | | |
| PC (38:5) | ↓ | 0.48 | ↑ | 1.22 | | | | |
| PC (32:0) | ↓ | 0.45 | ↑ | 1.32 | ↑ | 1.44 | | |
| PC (40:6) | ↓ | 0.53 | ↑ | 1.27 | | | | |
| PC (36:3) | ↓ | 0.38 | ↑ | 1.20 | ↑ | 1.30 | | |
| PC (36:2) | ↓ | 0.46 | ↑ | 1.29 | | | | |
| PC (34:2) | ↓ | 0.41 | ↑ | 1.29 | ↑ | 1.38 | | |
| PC (40:5) | ↓ | 0.50 | ↑ | 1.19 | ↑ | 1.22 | | |
| PS (40:5) | ↓ | 0.36 | | | | | | |
| PS (40:4) | ↓ | 0.43 | | | | | | |
| PS (36:2) | ↓ | 0.42 | | | | | | |
| PEo(40:7)/PEp(40:6) | ↓ | 0.74 | ↑ | 1.17 | ↑ | 1.25 | | |
| PEo(40:6)/PEp(40:5) | ↓ | 0.50 | | | ↑ | 1.32 | | |
| SM (d42:2) | ↑ | 1.48 | | | | | ↓ | 0.71 |

**Reference**

**1.** Kolde, R., 2019. Pheatmap: Pretty Heatmaps. R Package Version 1012. https. CRANR-project. org/package= pheatmap [Google Scholar].

2. Team, R.C., 2013. R: A language and environment for statistical computing. Vienna, Austria.

Wickham, H., 2016. ggplot2: elegant graphics for data analysis. springer.
